# Alterations in Energy Metabolism, Neuroprotection and Visual Signal Transduction in the Retina of Parkinsonian, MPTP-Treated Monkeys

**DOI:** 10.1371/journal.pone.0074439

**Published:** 2013-09-05

**Authors:** Laura Campello, Julián Esteve-Rudd, Roque Bru-Martínez, María Trinidad Herrero, Emiliano Fernández-Villalba, Nicolás Cuenca, José Martín-Nieto

**Affiliations:** 1 Departamento de Fisiología, Genética y Microbiología, Facultad de Ciencias, Universidad de Alicante, Alicante, Spain; 2 Departamento de Agroquímica y Bioquímica, Facultad de Ciencias, Universidad de Alicante, Alicante, Spain; 3 Instituto Multidisciplinar para el Estudio del Medio Ramón Margalef, Universidad de Alicante, Alicante, Spain; 4 Departamento de Medicina, Facultad de Ciencias de la Salud, Unidad de Neurociencia Clínica y Experimental (NiCE), Centro de Investigación Biomédica en Red sobre Enfermedades Neurodegenerativas (CIBERNED), Universitat Jaume I, Castellón, Spain; 5 Departamento de Anatomía Humana y Psicobiología, Facultad de Medicina, NiCE-CIBERNED, Universidad de Murcia, Murcia, Spain; Massachusetts Eye & Ear Infirmary, Harvard Medical School, United States of America

## Abstract

Parkinson disease is mainly characterized by the degeneration of dopaminergic neurons in the central nervous system, including the retina. Different interrelated molecular mechanisms underlying Parkinson disease-associated neuronal death have been put forward in the brain, including oxidative stress and mitochondrial dysfunction. Systemic injection of the proneurotoxin 1-methyl-4-phenyl-1,2,3,6-tetrahydropyridine (MPTP) to monkeys elicits the appearance of a parkinsonian syndrome, including morphological and functional impairments in the retina. However, the intracellular events leading to derangement of dopaminergic and other retinal neurons in MPTP-treated animal models have not been so far investigated. Here we have used a comparative proteomics approach to identify proteins differentially expressed in the retina of MPTP-treated monkeys. Proteins were solubilized from the neural retinas of control and MPTP-treated animals, labelled separately with two different cyanine fluorophores and run pairwise on 2D DIGE gels. Out of >700 protein spots resolved and quantified, 36 were found to exhibit statistically significant differences in their expression levels, of at least ±1.4-fold, in the parkinsonian monkey retina compared with controls. Most of these spots were excised from preparative 2D gels, trypsinized and subjected to MALDI-TOF MS and LC-MS/MS analyses. Data obtained were used for protein sequence database interrogation, and 15 different proteins were successfully identified, of which 13 were underexpressed and 2 overexpressed. These proteins were involved in key cellular functional pathways such as glycolysis and mitochondrial electron transport, neuronal protection against stress and survival, and phototransduction processes. These functional categories underscore that alterations in energy metabolism, neuroprotective mechanisms and signal transduction are involved in MPTP-induced neuronal degeneration in the retina, in similarity to mechanisms thought to underlie neuronal death in the Parkinson’s diseased brain and neurodegenerative diseases of the retina proper.

## Introduction

Parkinson disease (PD) is one of the most common progressive neurodegenerative disorders in our society, affecting over 1% of the aged people in Western countries and with a current estimated prevalence in the world of around 4.5 million persons [1]. Collected under the term parkinsonism, the symptoms exhibited by patients with this disease include resting tremor, rigidity, postural instability and bradykinesia [2]. The causal origin of PD is unknown in most cases, and treatments available today, based on the administration of levodopa and dopamine analogues, although substantially ameliorate clinical symptoms, do not alter the course of disease [3]. This is characterized by the massive, progressive and irreversible degeneration of dopaminergic cell bodies located in the midbrain substantia nigra and their axon terminals in the striatum, and by their cytoplasmic accumulation of typical protein fibrillar inclusions, named Lewy bodies [2,4]. This leads to a decrease in the substantia nigra of the levels of Tyr hydrolase (TH), the characteristic enzyme of dopaminergic neurons, and a decrease of the neurotransmitter dopamine in the striatum. Although the etiology of parkinsonism is still largely unknown, it is nowadays considered as a multifactorial disease beyond any doubt. Hence, although considerable progress has been made on the identification of genes associated with the development of parkinsonism, the action of environmental factors, mostly unknown, is recognized to be the cause of most cases of common (sporadic or idiopathic) PD [1,5,6]. In this context, some neurotoxins are known to induce the selective destruction of dopaminergic neurons, such as 1-methyl-4-phenyl-1,2,3,6-tetrahydropyridine (MPTP), 6-hydroxydopamine and a variety of metals and pesticides, such as rotenone, whose administration to monkeys and rodents has allowed the generation of animal models of idiopathic PD [7–9] that add up to available rodent genetic models [10]. Extensive research on these experimental systems together with available PD patients has led to envision a series of molecular mechanisms as accounting for the pathophysiology of PD, including oxidative stress, mitochondrial dysfunction and excitotoxic damage [5,6,11,12], among others including mutations in genes encoding proteins with a neuroprotective role, such as parkin [2,5]. In this context lies the repeated finding in PD patients of decreased activity of complex I of the electron transport chain and, recently, of mitochondrial DNA deletions [13]. Hence, the chemical inhibition of complex I by systemically-administered MPTP, 6-hydroxydopamine or rotenone recapitulates dopaminergic neuron degeneration and associated neurological disability in experimental animals [6,7]. Also, the proposed prooxidant intracellular status generated by a cytoplasmic accumulation of dopamine, resulting from its defective vesicular storage, has been postulated to lead dopaminergic neurons to apoptosis [11,14].

The mammalian retina is the tissue with the most active metabolism in the organism and hence exhibiting the highest O_2_ consumption. Any impairment in mitochondrial activity, induced for instance by the above neurotoxic agents, is thus expected to greatly affect the retina as part of the central nervous system (CNS). A growing body of experimental evidence has accumulated concerning visual dysfunction in the retina of parkinsonian patients including loss of visual acuity, contrast sensitivity, colour discrimination and motion perception [15–17]. The relevance of macaques treated with MPTP [18,19] or 6-hydroxydopamine [20] as a model of parkinsonism in the retina was initially supported by a series of studies highlighting a number of dysfunctional features detectable by electroretinogram and visual evoked potential recordings, which are similar to those reported in PD patients. These deficits have been correlated by our group with a series of morphological alterations occurring in the retina of MPTP-treated monkeys, where loss of TH immunoreactivity in the substantia nigra was accompanied by the degeneration of both retinal dopaminergic neurons and their postsynaptic AII amacrine cells, together with synapses formed among these neuronal subtypes in the inner plexiform layer [21]. More recently, we have also characterized the cellular and functional impairments taking place in the retina of rotenone-treated rats, where damage extended not only to dopaminergic neurons, but also to photoreceptors and their synaptic connectivity [22]. Deficiencies occurring at the molecular level in the parkinsonian retina are, however, essentially unknown, as are the particular mechanisms by which MPTP or rotenone elicits the degeneration of dopaminergic cells and other retinal neurons. In this work we have investigated the alterations in the retinal proteome taking place in the MPTP-treated monkey model of PD, in order to provide insight into the molecular mechanisms underlying neuronal degeneration in the retina under parkinsonism. Most of the proteins identified as differentially expressed in parkinsonian monkeys were found to be downregulated, and their functions were related to cellular energy metabolism, neuroprotection against stress and visual signal transduction.

## Materials and Methods

### Biological Material and Ethics Information

All studies were carried out in accordance with the guidelines promulgated by the European Convention for the Protection of Vertebrate Animals used for Experimental and other Scientific Purposes of the Council of Europe (no. 123; June 15th, 2006), and following the Code of Ethics of the European Directive 2010/63/EU and the U.S. National Institutes of Health (NIH). All protocols and animal handling procedures used were approved by the bioethics research committee of the Universidad de Murcia [23]. Long-tailed macaques (*Macaca fascicularis*) were imported from R.C. Hartelust B.V. (Tilburg, the Netherlands) and housed at the animal care facility of the Universidad de Murcia under supervision by veterinarians and technicians skilled in the health care and maintenance of non-human primates. The animals were maintained in cages of 65 x 75 x 95 cm in a primate house under temperature (25^°^C) and humidity (50%) controlled conditions and a 12 h light/12 h dark cycle (lights on at 8:30 AM), and they had free access to special food (Masuri primate diet; Scientific Dietary Services, UK), fresh fruit and water. Even though animals were housed in partners, the arrangement of cages allowed each individual to have visual contacts and interaction with monkeys housed in adjacent cages. Animals used in the present work had not been utilized for any prior research.

Experiments were performed on three adult macaques (4-6 kg; both sexes) that were rendered parkinsonian upon treatment with MPTP hydrochloride (Sigma; 0.3 mg/kg i.v. for several months, 1 injection every 2 weeks). Monkeys were injected in the saphenous vein under gentle restraint and were never given levodopa or dopaminergic agonists. The number of MPTP doses administered was adjusted according to each animal’s susceptibility to this compound, until a stable parkinsonian syndrome was reached [21,23]. Three additional, age-matched monkeys were kept without injections under the same conditions as the three treated, and used as control subjects. The total dose of MPTP and the time necessary to obtain sustained parkinsonian features ranged from 20 to 24 mg and from 26 to 28 weeks, respectively. This protocol represents a reproducible MPTP cumulative dosing regimen that leads to the first appearance of parkinsonian clinical signs after 6 ± 1 injections. Animal behaviour was assessed both in their home cages and in special observation cages for filming. One special cage, measuring 1 m^3^, was designed so that an independent camera located in front of the cage, in a special space separated from the animals with methacrylate, could perform independent recordings from each animal. The parkinsonian syndrome was independently analyzed by several scientists accustomed to evaluating motor disabilities in monkeys, which was carried out by coaxing the animals to perform various tasks upon offering them appetizing fruits. The degree of parkinsonism was scored twice weekly, over a 10 min observation period with respect to control animals, on the basis of a validated parkinsonian macaque clinical motor scale ranging from 0-2 (normal) to 25 (maximum severity) [24]. This scale covers eight features of motor disability induced by MPTP: spontaneous activity (0-5), bradykinesia (0-3), tremor duration (0-3), tremor intensity (0-3), posture (0-3), balance (0-2), freezing (0-3) and feeding (0-3).

No animal was sacrificed solely for the purpose of the research reported here, but instead other body parts were harvested by different researchers for a range of unrelated experiments, and their suffering was minimized at all times during this procedure [23]. The monkeys were anesthetized with ketamine (10 mg/kg i.m.) and then administered a lethal injection of pentobarbital (50 mg/kg i.p.). Eyeballs were then enucleated, and the neural retina was dissected free from the retinal pigment epithelium, frozen in liquid N_2_ for transport to the Universidad de Alicante and stored at -80^°^C until use.

### Protein Extraction and Processing

Retinal tissue samples were thawed at room temperature and homogenized in lysis buffer (20 mM Hepes pH 7.9, 10% glycerol, 10 mM KCl, 0.4 M NaCl, 0.1% Nonidet P-40 and 2 mM DTT) supplemented with protease inhibitors (1 mM PMSF, 10 μM leupeptin, 0.3 μM aprotinin, 100 μM benzamidine). After incubation on ice for 15-20 min with occasional gentle shaking, samples were centrifuged at 13,000 rpm for 10 min at 4 °C. The supernatant, containing solubilized proteins, was aliquoted and stored at -80^°^C. Protein concentration was quantified spectrophotometrically at 595 nm using the Bradford reagent (Sigma) and BSA as a standard.

Solubilized proteins (200 μg for analytical gels, 900 μg for preparative gels) were preincubated in 0.2 mg/l sodium deoxycholate (Sigma) for 15 min on ice. Then, they were precipitated by addition of trichloroacetic acid to 6% and incubation for 30 min on ice, and recovered by centrifugation at 16,000×g for 15 min at 4 °C. The pellet obtained was washed twice with 1.5 ml of 80% acetone (precooled at -20^°^C), centrifuging each time at 16,000×g for 15 min at 4 °C. Then the pellet was dried for 15 min at room temperature, and dissolved by vigorous stirring in 100 μl of 2D lysis buffer composed of 7 M urea, 2 M thiourea, 4% 3-[(3-cholamidopropyl) dimethylammonio]-1-propanesulfonate (CHAPS) and 30 mM Tris base. The proteins were purified with the 2-D Clean-Up kit from GE Healthcare (Buckinghamshire, UK) and finally dissolved in 40 μl of 2D lysis buffer by vigorous shaking and sonication for 10 min. Protein concentration was quantified using the RC-dC Protein Assay from Bio-Rad (Hercules, CA).

### Fluorescent Labelling of Proteins

The pH of samples (50 μg of protein) for DIGE was adjusted to 8.5-9.0 by adding 1-2 μl of 100 mM NaOH. Then cyanine fluorescent dyes (GE Healthcare) were conjugated to solubilized proteins via N-hydroxysuccinimidyl linkages following the manufacturer’s minimal labelling protocol. Briefly, the parkinsonian or control protein sample was taken to a volume of 18 μl with 2D lysis buffer and labelled alternately with Cy3 (green) or Cy5 (red) by addition of 1 μl of 0.4 mM fluorophore in N,N-dimethylformamide. Separately, an internal standard was prepared consisting of a pool of identical amounts of protein from the six monkey retinal samples labelled with Cy2. Reactions were carried out for 30 min on ice in the dark, and quenched by addition of 1 μl of 10 mM Lys and further 10 min incubation. For each gel, 50 μg of control group sample and 50 μg of MPTP group sample, each labelled with a different dye, was mixed with 50 μg of Cy2-labelled internal standard, to obtain a 65 μl mixture containing 150 μg of protein. Then DIGE sample buffer containing 7 M urea, 2 M thiourea, 2% CHAPS, 2% IPG buffer pH 4-7 (GE Healthcare) and 130 mM DTT was added to a final volume of 130 μl, and the mixture was incubated on ice for further 15 min. To avoid biasing due to the dye, half of the samples of each experimental group were labelled with Cy3 and the other half with Cy5 [25].

### 2D Gel Electrophoresis

For IEF in the first dimension 18 cm-long Immobiline DryStrips (GE Healthcare) were used, containing immobilized ampholytes forming a linear pH 4-7 gradient. The strips were rehydrated in 350 μl of a buffer containing 7 M urea, 2 M thiourea, 2% CHAPS, 2% IPG buffer pH 4-7, 18 mM DTT and traces of bromphenol blue, for 16 h at room temperature covered with mineral oil. The samples were applied onto the strips following a cup loading protocol, and IEF was performed at room temperature in an Ettan IPGphor II unit (GE Healthcare) at 50 mA per IPG strip, according to the following program: fast gradient to 300 V for 3 h, linear gradient to 1,000 V for 6 h, linear gradient to 8,000 V for 3 h, and fast gradient to 8,000 V for 4 h, totalling 40,000 V·h at the end of the run.

After IEF, the IPG strips were incubated with shaking for 15 min in equilibration buffer I (6 M urea, 30% glycerol, 2% SDS, 50 mM Tris-HCl pH 8.8, 1% DTT), and then for further 15 min in equilibration buffer II (buffer I in which DTT was replaced by 1.25% iodoacetamide). Thereafter, they were individually applied onto the surface of a vertical 12.5% polyacrylamide gel and sealed with 0.5% agarose dissolved in electrophoresis buffer (25 mM Tris-HCl pH 8.3, 192 mM Gly, 0.1% SDS) with traces of bromphenol blue. SDS-PAGE Broad Range or All Blue Precision Plus Protein (Bio-Rad) molecular weight standards were loaded by each end of the strip, and the second-dimension electrophoresis was carried out in a DALTsix Ettan unit (GE Healthcare) at a constant current of 10 mA for 14-16 h, or 40 mA for 4-5 h.

### Gel Scanning and Staining

Following electrophoresis, signals from the three fluorophores in each gel were sequentially scanned on a Typhoon 9410 fluorescence imager (GE Healthcare) at a photomultiplier voltage of 500 V and a pixel size of 100 μm (254 dpi resolution). For Cy3, excitation and emission wavelengths used were 532 and 580 nm, respectively, and for Cy5 633 and 670 nm. Cy2 images were obtained at wavelengths of 488 and 520 nm.

DIGE gels were stained with silver nitrate, essentially as previously described [26]. Preparative gels were stained with colloidal Coomassie Blue according to established procedures [27], with some modifications. Briefly, gels were fixed in 50% ethanol and 2% phosphoric acid for 3 h and, after washing with milliQ H_2_O, incubated for 1 h in staining solution (3% phosphoric acid, 35% methanol, 100 mM ammonium sulfate). Then Coomassie Brilliant Blue G-250 solution in methanol (USB, Cleveland, OH) was added to a concentration of 0.65 g/l, and the gels were incubated for 14-16 h at room temperature. After destaining with milliQ H_2_O, they were stored in 5% acetic acid at 4 °C. Gels were digitized on an UVIdoc Documentation System (UVItec, Cambridge, UK).

### MS Analyses and Database Search for Protein Identification

With the aim of identifying the proteins detected in the retina as differentially expressed in control vs. MPTP-treated monkeys, the spots were manually excised from Coomassie Blue-stained preparative polyacrylamide gels using a manual spot picker (Gel Company, Tübingen, Germany) with a 1.5 mm diameter picker head, and processed for trypsin digestion by using an Investigator Progest protein workstation (Genomic Solutions, Cambridgeshire, UK). The protein spots were washed sequentially in this system with 25 mM ammonium bicarbonate and then with H_2_O, and thereafter subjected to reduction with 10 mM DTT and alkylation with 100 mM iodoacetamide in 50 mM ammonium bicarbonate. The proteins were then digested with modified porcine trypsin from Promega (Madison, WI), in 25 mM ammonium bicarbonate for 6-7 h at 37 °C. The resulting peptides were sequentially extracted with ammonium bicarbonate, then with 70% acetonitrile, and finally with 1% formic acid.

For MALDI-TOF MS analysis, the peptides were dried under vacuum in a SpeedVac system for at least 1 h at 60^°^C. Then they were purified and concentrated on Zorbax 300SB-C18 silica gel tips (Agilent Technologies, Santa Clara, CA), and redissolved in 5 μl of a 0.1% trifluoroacetic acid: acetonitrile mixture (2:1). One μl of sample was spotted onto a MALDI plate (MTP AnchorChip 400/384 TF, from Bruker Daltonics, Bremen, Germany) together with 1 μl of 2,5-dihydroxybenzoic acid matrix dissolved in the same trifluoroacetic acid: acetonitrile mixture. Prior to analysis, the sample was allowed to dry on the matrix and an external calibration of the MALDI-TOF Autoflex mass spectrometer was performed using the PeptideMix peptide standard (both from Bruker Daltonics). The peptides in each sample were ionized with a 337-nm N_2_ laser, and mass spectra were acquired in the positive ion mode at a reflectron voltage of 20 kV by averaging 30 laser shots. The range of masses considered was 800-3500 Da, suitable to cover in general the majority of peptides from trypsin digestion.

For LC-MS/MS, tryptic peptides were SpeedVac-dried as above and redissolved in 15 μl of 0.1% formic acid. LC-MS/MS analysis was performed using an Agilent 1100 nano-high-performance liquid chromatography system coupled to a mass spectrometer equipped with an XCTplus ion trap and an electrospray ionization source (Agilent Technologies). After concentration and desalting of peptides by employing a Zorbax 300SB-C18 column (0.3 mm × 5 mm, 5 μm) at a flow rate of 0.3 μl/min, they were separated on a Zorbax 300SB-C18 analytical column (75 μm × 15 cm, 3.5 μm) using a linear gradient of 5-45% acetonitrile in 0.1% formic acid at a constant flow of 0.3 μl/min, for 50 min. For acquisition of mass and mass/mass spectra, the parameters previously indicated [28] were used, with some modifications. Briefly, the spectra were obtained using the standard enhanced and UltraScan modes, at 26,000 and 8,100 m*/z* per second respectively. Parameters included a ionization potential of 1.8 kV and an ICC smart target (number of ions in the trap before acquisition) of 500,000 or a maximum accumulation time of 150 ms. MS/MS spectra analyses were performed using automated switching with a preference for +2 charged ions, a threshold of 105 counts and a 1.3 kV fragmentation amplitude.

Peptide mass fingerprints obtained from MALDI-TOF MS analysis were used to interrogate the NCBInr polypeptide database by employing the Mascot search engine [29]. Parameters were configured as follows: maximum trypsin missed cleavages, 1; fixed modification, Cys carbamidomethylation; variable modification, Met oxidation; peptide mass tolerance, ±100 ppm; mass values, monoisotopic, and peptide charge state, 1+. Molecular weight s earch (Mowse) scores were calculated by comparison of search results against estimated random match population, and reported as -10 × log (P) where P is the absolute probability [30]. Only those identifications that met the following criteria were considered as valid: significant (>80) Mowse score, ≥4 peptides identified and sequence coverage >25%.

The MS/MS spectra obtained from LC-MS/MS analysis were used to search the Swiss-Prot polypeptide database with the Spectrum Mill Proteomics Workbench software rev. A.03.03 (Agilent Technologies). Parameters were set as: maximum missed cleavages, 2; fixed modification, Cys carbamidomethylation; variable modifications, Met oxidation, Asn deamidation and pyro-Glu formation; and fragment mass tolerance, ±0.7 and ±0.6 Da for parental and fragment ions, respectively. Only those identifications that met the following criteria were considered as valid: scores given by the Spectrum Mill software, >8 to each peptide and >20 to the protein, and sequence determined for at least 2 different peptides. The automatic validation of each protein and peptide was verified manually to avoid false positives. The raw data (mzXML) and peak lists (*. PKL) are available as a 1.4 Gb compressed file upon request to the corresponding author.

### Western Blotting

Retinal proteins were extracted and subjected to immunoblotting analysis as previously described [31,32]. Proteins (25 or 75 μg/lane) were resolved by SDS-PAGE on 5–20% polyacrylamide-gradient gels and electrotransferred to Hybond-P PVDF membranes (GE Healthcare). These were probed at 4 °C overnight with mouse monoclonal antibodies to S-arrestin (clone SCT-128; provided by W. Clay Smith) at a 1:1,000 dilution, calbindin D28k (clone CB-955; Sigma) at a 1:500 dilution, to NDP kinase α (clone KM1121; Kamiya Biomedical Company, Seattle, WA) at a 1:300 dilution, or to β-actin (clone AC-15; Sigma) at a 1:20,000 dilution. Thereafter, they were incubated at room temperature for 1 h with horseradish peroxidase-conjugated goat anti-mouse IgG (Pierce, Rockford, IL) at a 1:10,000 dilution. Detection was performed by ECL using the SuperSignal West Dura system (Pierce).

### Statistical and Quantitative Analyses

The spot staining pattern of DIGE gels was analyzed using the Progenesis SameSpots software v. 3.0 (Nonlinear Dynamics, Newcastle, UK). Equivalent spots across gels were matched, and images from control and parkinsonian monkeys were separately grouped. Spots outlined by the program were also visually inspected and manually edited when appropriate. The amount of protein present in each spot was densitometrically measured and its volume was calculated by integration of the optical density over the spot’s area. Then normalized volumes (NV) were obtained by dividing it by the total volume over the whole set of gel spots (yielded by the internal standard, labelled with Cy2), and the NV values of each spot were separately averaged for control and MPTP-treated monkeys. The fold change in protein expression was calculated as the ratio between the higher and the lower of each pair of values, and a minus sign (-) was added when the lower was that of the treated group. The processing of numerical data was performed using Microsoft Excel, and statistical analyses were carried out using the Prism v. 5 program from GraphPad Software (San Diego, CA). Significance of differences between treatments was evaluated using the non-parametric Student’s *t*-test with a confidence interval of 95%. In DIGE experiments the differences were additionally evaluated by analysis of variance (ANOVA), and only spots yielding a P value lower than 0.05 in both tests were considered for further analysis.

Autoradiography films from Western blots were digitized using an ImageScanner in transparency mode at 300 dpi and 16 bits grey scale, and image acquisition was performed using the LabScan v. 5.0 software (both from GE Healthcare). Densitometric quantitation of protein bands was accomplished using ImageJ 1.42q [33], and values obtained for each protein were normalized to β-actin levels. For each quantified protein we analyzed the results of at least two independent experiments, each containing a minimum of three biological replicates.

## Results

Three eyes were used for this study each from a different macaque that had been chronically treated with MPTP and who had developed a stable and persistent parkinsonian syndrome, from moderate to severe as judged from its motor alterations. Two of them exhibited a severe parkinsonism (motor disability, 16/25) and the third moderate parkinsonian symptoms (motor disability, 13/25). These monkeys displayed bradykinesia/akinesia, rigidity, freezing phenomena, action tremor and paradoxical kinesias, as well as abnormal vertical and horizontal saccadic ocular movements. They also showed postural disturbances (trunk and limb flexion) and balance alterations with occasional falls. Three additional, age-matched monkeys kept without injections under the same conditions as the three treated were used as control subjects (motor disability, 0-2/25). After sacrifice, their eyes were enucleated and their neural retinas dissected. Monkeys treated in the same fashion and exhibiting comparable motor scores displayed a substantial reduction of TH immunoreactivity in both the substantia nigra and the retina, resulting in the degeneration of dopaminergic cell bodies and dendritic plexi in both tissues [21].

### Analysis of Differential Protein Expression

**Figure 1:**
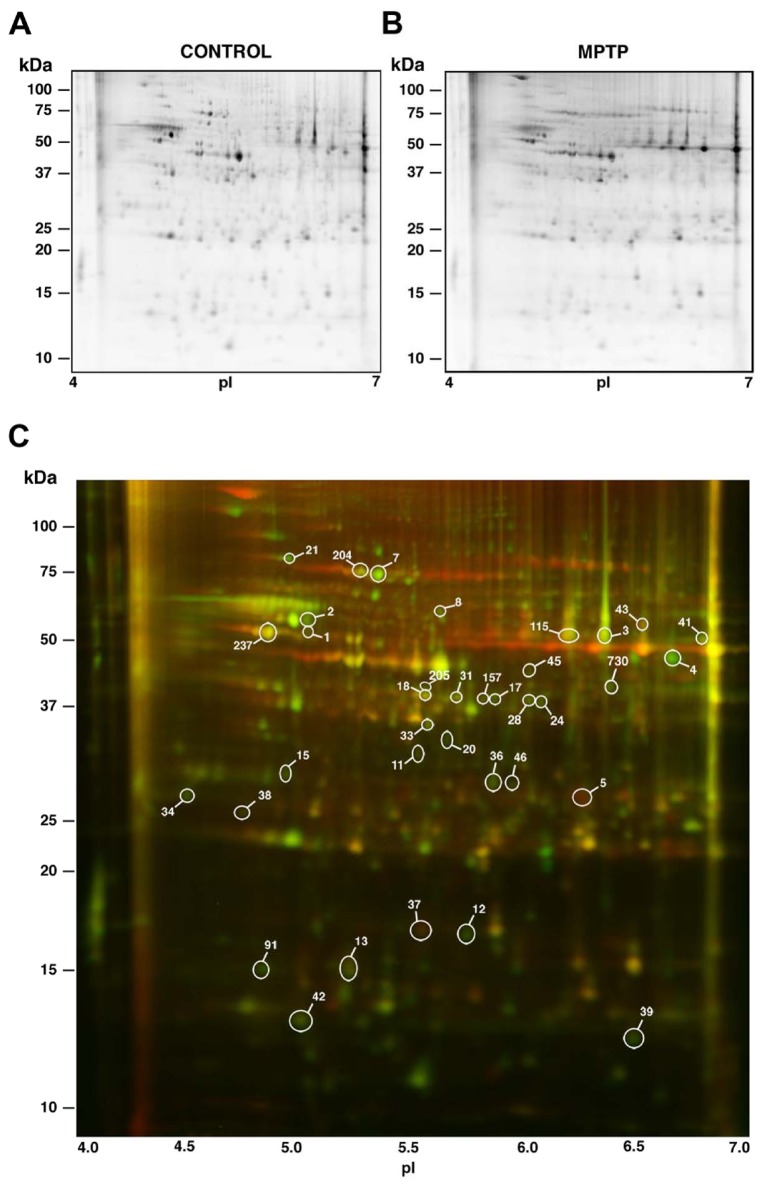
DIGE-resolved polypeptides from the retina of control and MPTP-treated monkeys. **A**. Control monkey. **B**. MPTP-treated monkey. **C**. Merged image resulting from superimposition of A (proteins labelled in green) and B (proteins in red). Yellow spots correspond to polypeptides with similar expression levels in both samples. Differentially-expressed spots are circled, and the indicated numbering was subsequently used throughout tables in this work.

Differences in the abundance of particular proteins in the retina of parkinsonian vs. control monkeys were screened by means of 2D DIGE technology. A preliminary 2D gel was run in the pH range 3-10, where most detected proteins were observed to fall within the pI interval 4-7. Therefore, this pI range was subsequently used for the first-dimension IEF. For the second dimension regular SDS-PAGE was carried out at a fixed, 12.5% polyacrylamide concentration. Thereafter, the 2D gels were scanned with a Typhoon 9410 Variable Mode Imager and the spot maps obtained were bioinformatically analyzed using the Progenesis SameSpots software. A representative DIGE gel is shown in Figure 1, where separate images corresponding to the proteins from a control parkinsonian monkey retina (labelled in green) and from an MPTP-treated subject (labelled in red) are shown in Figure 1A and 1B, respectively. The merged image is shown in Figure 1C, where yellow spots represented proteins with similar expression levels in both samples and were thus not considered.

Over 700 discrete spots were clearly resolved on 2D DIGE gels. The electrophoretic pattern obtained was consistent for retinal extracts from individuals of the same group (i.e. control or MPTP-treated monkeys), although a certain variability was observed attributable to the fact that the animals studied were not isogenic. A bioinformatic analysis revealed that quantitative differences existed for a total of 36 spots between the two experimental groups, whose positions in 2D gels are numerically labelled in Figure 1C. These spots were selected for their variation exceeding an arbitrary threshold of 1.4-fold increase (40%) or decrease (29%) in their expression levels and for differences being concluded to be statistically significant from both the Progenesis SameSpots software and Student’s *t*-test. In particular, 34 spots exhibited a decrease of expression above 1.4-fold in parkinsonian monkeys and 2 spots a higher than 1.4-fold increase with respect to controls (Table 1). Among the 34 spots whose normalized volume had diminished, the greatest decrease was observed for spot no. 2, whose abundance was reduced by 2.97-fold (to 33.7%). On the other hand, spots no. 5 and 37 had increased their normalized volumes, by 2.52 and 1.41-fold respectively, in the retina of MPTP-treated monkeys. Qualitative differences, i.e. spots that were consistently absent in either of the two groups and present in the other, were not detected.

**Table 1:** Polypeptides differentially expressed in the retina of MPTP-treated versus control monkeys.

**Spot no.**	**Normalized volume**		**Fold change^a^**	**P^b^**
	**Control**	**MPTP**		
1	0.994 ± 0.296	0.514 ± 0.094	-1.93	0.0261*
2	1.922 ± 0.936	0.648 ± 0.100	-2.97	0.0352*
3	1.370 ± 0.357	0.486 ± 0.174	-2.82	0.0043**
4	1.436 ± 0.333	0.534 ± 0.401	-2.69	0.0135*
5	0.635 ± 0.176	1.600 ± 0.606	2.52	0.0223*
7	1.382 ± 0.603	0.559 ± 0.197	-2.47	0.0410*
8	1.427 ± 0.588	0.585 ± 0.336	-2.44	0.0474*
11	1.668 ± 0.653	0.745 ± 0.222	-2.20	0.0366*
12	1.204 ± 0.289	0.746 ± 0.155	-1.61	0.0407*
13	1.361 ± 0.281	0.647 ± 0.226	-2.10	0.0074**
15	1.393 ± 0.181	0.673 ± 0.256	-2.07	0.0037**
17	1.438 ± 0.467	0.717 ± 0.165	-2.01	0.0269*
18	1.415 ± 0.472	0.710 ± 0.104	-1.99	0.0269*
20	1.185 ± 0.345	0.607 ± 0.287	-1.95	0.0421*
21	1.539 ± 0.479	0.792 ± 0.111	-1.94	0.0228*
24	1.376 ± 0.262	0.747 ± 0.087	-1.84	0.0039**
28	1.330 ± 0.323	0.733 ± 0.131	-1.81	0.0142*
31	1.385 ± 0.386	0.799 ± 0.097	-1.73	0.0257*
33	1.247 ± 0.363	0.746 ± 0.120	-1.67	0.0395*
34	1.540 ± 0.267	0.934 ± 0.221	-1.65	0.0128*
36	1.391 ± 0.316	0.845 ± 0.174	-1.65	0.0232*
37	0.949 ± 0.237	1.341 ± 0.104	1.41	0.0466*
38	1.554 ± 0.302	0.946 ± 0.376	-1.64	0.0453*
39	1.420 ± 0.345	0.871 ± 0.140	-1.63	0.0256*
41	1.374 ± 0.089	0.871 ± 0.277	-1.58	0.0135*
42	1.345 ± 0.273	0.861 ± 0.211	-1.56	0.0309*
43	1.265 ± 0.141	0.828 ± 0.162	-1.52	0.0066**
45	1.329 ± 0.131	0.931 ± 0.223	-1.43	0.0220*
46	1.388 ± 0.245	0.993 ± 0.124	-1.40	0.0279*
91	1.277 ± 0.358	0.816 ± 0.072	-1.56	0.0489*
115	1.463 ± 0.424	0.676 ± 0.353	-2.17	0.0430*
157	1.482 ± 0.528	0.864 ± 0.235	-1.71	0.0287*
204	1.204 ± 0.299	0.766 ± 0.278	-1.57	0.0192*
205	1.117 ± 0.221	0.711 ± 0.236	-1.57	0.0457*
237	1.197 ± 0.321	0.790 ± 0.204	-1.51	0.0382*
730	1.370 ± 0.409	0.743 ± 0.080	-1.84	0.0237*

a Calculated by dividing the higher by the lower number in each pair of NV values, and preceded by a minus (-) symbol when the lower was that of the MPTP-treated sample.

^b^ Calculated for Student’s *t*-test (*P <0.05, significant; **P <0.01, highly significant).

### Identification of Differentially-Expressed Proteins

From preparative 2D gels stained with colloidal Coomassie Blue a series of proteins were recovered that exhibited altered amounts in the parkinsonian monkey retina when compared to controls. A total of 29 spots were selected on the basis of their unequivocal visualization and localization in the preparative gel, and following excision and trypsin digestion they were subjected to MS analysis. Eighteen spots were successfully identified by means of peptide mass fingerprinting and/or MS/MS search analyses by interrogation of the NCBInr and Swiss-Prot polypeptide databases, respectively, and the characteristics of the corresponding proteins are summarized in Table 2. In instances where these databases did not contain any matching *M. fascicularis* protein sequence (most cases), the identification was carried out on the basis of its homology to the human (*Homo sapiens*), rhesus macaque (*Macaca mulatta*) or chimpanzee ( *Pan* *troglodytes* ) ortholog sequence. Table 2 includes, in addition to protein names, their accession numbers in the corresponding database (NCBInr and/or Swiss-Prot) and the species to whom such sequence belongs. Also indicated for each protein in Table 2 are its pI and *M*r values, both experimental (calculated from its migration position in 2D gels) and theoretical (from its amino acid sequence in the database), together with the MS method(s) allowing its identification. Some spot pairs corresponded to the same protein, in particular 3 and 115 (S-arrestin), 13 and 18 (DDAH1), and 20 and 237 (γ-enolase), which represented isoelectric variants derived from proteolytic processing or other post-translational modifications changing their intrinsic charge and/or mass. For some of the identified proteins significant differences were found between their experimental and theoretical pI (e.g. spots 1 and 37) or *M*r (e.g. spots 13 and 20) values, which could be attributed to the polypeptide excised from the gel harbouring covalent modifications occurring post-translationally (e.g. phosphorylation, glycosylation, etc.) or “accidentally” during sample handling (e.g. oxidation), or else to its encoding mRNA undergoing alternative splicing. For identifications carried out by peptide mass fingerprinting followed by database screening using the Mascot search engine, Table 2 shows each protein’s Mowse score, taken as a confidence index, the number of peptides whose mass was represented in the fingerprint, and the sequence coverage. Mowse scores for this group of proteins ranged between 133 and 294, the sequence coverage between 53 and 73%, and the number of matching peptides between 15 and 21. Table S1 gives for each protein spot the number of peptide masses obtained by MALDI-TOF used for database screening, the number of masses matching amino acid segments of the identified protein, and sequences of the latter. For identifications carried out by LC-MS/MS, the score yielded by the Spectrum Mill software is given in Table 2, together with the percentage of sequence coverage and the number of identified peptides. For this group of proteins, which constituted the vast majority, the MS/MS search scores varied between 17.43 and 378.20, the sequence coverage between 6 and 58% and the number of peptides between 2 and 21. Those with the lowest values for these three parameters, i.e. spots no. 20 and 34, were identified by *de novo* sequencing using the Sherenga algorithm [34], illustrated in Figure S1. The sequences of the different peptides identified in each protein spot by means of LC-MS/MS or *de novo* sequencing using Sherenga are shown in Table S2. The corresponding score, charge, scored peak intensities (SPI) and monoisotopic mass of each identified peptide precursor are also indicated in Table S2.

**Table 2:** Identification by mass spectrometry of proteins differentially expressed in the retina of parkinsonian monkeys.

**Spot no.**	**Protein**	**Accession no. ^a^**	**pI exp. / pI theor. ^b^**	***M*r exp. / *M*r theor. ^b^**	**Analysis method^b^**	**MALDI-TOF^c^ Score / Sequence coverage / Number of peptides**	**LC-MS/MS^d^ Score / Sequence coverage / Number of peptides**
1	α-Enolase	P06733 (S, Hs), NP_001419 (N, Hs)	4.75/6.99	48.2/47.1	LC-MS/MS, MALDI-TOF	133/53/16	244.30/39/12
2	ATP synthase, subunit β	P06576 (S, Hs)	4.75/5.00	52.4/51.8	LC-MS/MS		378.20/52/21
3	S-Arrestin	P10523 (S, Hs)	6.25/6.14	51.5/45.1	LC-MS/MS		67.24/10/5
5	β-Crystallin B2	XP_001098886 (N, Mm)	6.14/6.54	27.7/23.2	MALDI-TOF	261/60/15	
7	Heat shock 70 kDa protein 8 (HSC70)	P11142 (N, Hs)	5.08/5.37	75.0/70.8	LC-MS/MS		65.84/8/4
12	Stathmin	P16949 (N, Hs)	5.53/5.77	16.5/17.2	LC-MS/MS		31.58/15/2
13	N^G^,N^G^-Dimethylarginine dimethylaminohydrolase 1 (DDAH1)	O94760 (S, Hs)	4.93/5.53	15.2/30.1	LC-MS/MS		152.41/34/8
18	DDAH1	O94760 (S, Hs), NP_036269 (N, Hs)	5.32/5.53	36.9/30.1	LC-MS/MS, MALDI-TOF	171/63/16	218.67/58/13
20	γ-Enolase	P09104 (S, Hs)	5.44/4.91	32.2/47.1	LC-MS/MS		23.87/6^e^ / 2**^e^**
21	Glucose-regulated 78 kDa protein (GRP78)	P11021 (S, Hs)	4.67/5.01	82.1/72.3	LC-MS/MS		68.31/9/4
31	Glyceraldehyde 3-phosphate dehydrogenase (GAPDH)	P04406 (S, Hs)	5.49/8.58	37.4/35.6	LC-MS/MS		53.30/10/3
33	Inorganic pyrophosphatase (PPA1)	Q4R543 (S, Mf), XP_001164495 (N, Pt)	5.33/5.54	34.1/32.6	LC-MS/MS, MALDI-TOF	231/65/18	135.79/35/8
34	Calbindin	P05937 (S, Hs)	4.16/4.70	25.6/29.9	LC-MS/MS		17.43/8**^e^**/ 2**^e^**
37	Nucleoside diphosphate kinase B (NDPK B)	P22392 (S, Hs)	5.30/8.52	16.8/17.3	LC-MS/MS		44.49/25/3
42	Cytochrome c oxidase (COX), subunit 5A	Q53CF8 (S, Mm)	4.69/5.01	13.1/12.5	LC-MS/MS		36.40/16/2
91	γ-Synuclein	Q2PFW6 (S, Mf)	4.50/4.98	15.4/13.3	LC-MS/MS		96.86/49/6
115	S-Arrestin	P10523 (S, Hs)	6.06/6.14	50.6/45.1	LC-MS/MS		177.66/27/10
237	γ-Enolase	P09104 (S, Hs), AAV67362 (N, Mf)	4.54/4.91	48.5/47.1	LC-MS/MS, MALDI-TOF	294/73/21	204.93/44/13

a For each spot the protein identified is indicated together with its accession number in the interrogated polypeptide database (N, NCBInr; S, Swiss-Prot) and the corresponding primate species (Hs, human; Mf, long-tailed macaque; Mm, rhesus macaque; Pt, chimpanzee).

b The isoelectric point (pI) and relative molecular mass (*M*r) are also given, both experimental and theoretical, together with the MS method(s) used for its identification.

c The MOWSE score is indicated together with the fraction (%) of protein sequence covered by the identified peptides and the number of these represented in the mass fingerprint.

d The corresponding score is indicated together with the fraction (%) of sequence and the number of peptides identified.

e Peptides identified by *de novo* sequencing using the Sherenga algorithm.

**Figure 2:**
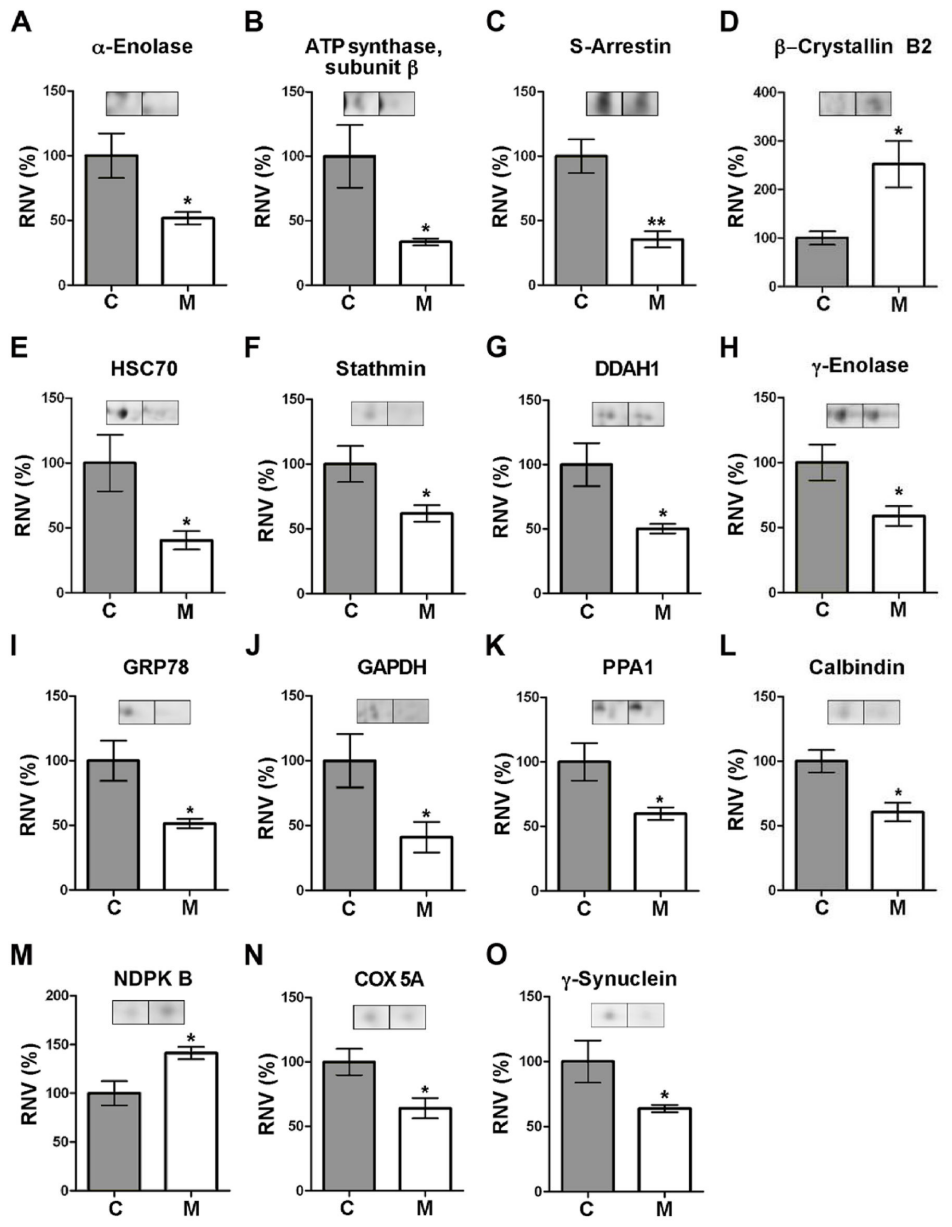
Levels of proteins differentially expressed in the retina of parkinsonian monkeys. **A**–**O**. Each plot shows the relative normalized volumes (RNV) for the identified polypeptides, obtained as the ratio between MPTP-treated (M) and control (C) NV values, taking the latter as 100%. Bars represent the average ± SEM (n = 3). The statistical significance obtained by Student’s *t*-test is indicated: * P <0.05; ** P <0.01. Representative spot pairs are also shown for each polypeptide, where the left spot corresponds to a control monkey and the right to a parkinsonian individual.

In total, a number of 15 different polypeptides were identified, whose differential expression levels are illustrated in Figure 2. These proteins are involved in a variety of functions within the cell. Table 3 summarizes, for each protein, its encoding gene and its accession number in the Gene Ontology (GO) database, where data on biological processes, cellular components and molecular functions associated with every known protein are compiled [35]. These are as follows:

**Table 3:** Function of differentially-expressed identified proteins.

**Spot no. ^a^**	**Protein^b^**	**Gene^b^**	**GO accession^c^**	**Biological process / Function^c^**
1	α-Enolase	*ENO1*	0006096; 0030308	Glycolysis; Negative regulation of cell growth
2	ATP synthase subunit β	*ATP5B*	0022904; 0005753	Respiratory electron transport chain; Mitochondrial H^+^-transporting ATP synthase complex
3, 115	S-Arrestin	*SAG*	0009586; 0007601	Rhodopsin mediated phototransduction; Visual perception
5	β-Crystallin B2	*CRYBB2*	0005212; 0007601	Structural constituent of eye lens; Visual perception
7	HSC70	*HSPA8*	0061077; 0006950	Chaperone-mediated protein folding; Response to stress
12	Stathmin	*STMN1*	0007019; 0033556; 0030154	Microtubule depolymerization; Intracellular signat transduction; Cell differentiation
13, 18	DDAH1	*DDAH1*	0016403; 0045429; 0017014	Dimethylargininase activity; Positive regulation of NO biosynthetic process; Protein nitrosylation
20, 237	γ-Enolase	*ENO2*	0006096; 0001917	Glycolysis; Photoreceptor inner segment
21	GRP78	*HSPA5*	0030968; 0051087; 0043066	ER unfolded protein response; Chaperone binding; Negative regulation of apoptotic process
31	GAPDH	*GAPDH*	0006096; 0051402; 0000226	Glycolysis; Neuron apoptotic process; Microtubule cytoskeleton organization
33	PPA1	*PPA1*	0004427	Inorganic diphosphatase activity
34	Calbindin	*CALB1*	0005509; 0048167; 0010842	Calcium ion binding; Regulation of synaptic plasticity; Retina layer formation
37	NDPK B	*NME2*	0004550; 0043066; 0010976	Nucleoside diphosphate kinase activity; Negative regulation of apoptotic process; Positive regulation of neuron projection development
42	COX subunit 5A	*COX5A*	0022904	Respiratory electron transport chain
91	γ-Synuclein	*SNCG*	0014059; 0005815	Regulation of dopamine secretion; Microtubule organizing centre

a Refers to that indicated in Figure 1C.

b The protein pertaining to each spot and the name of its encoding gene are indicated.

c Accession number(s) for each protein and most relevant associations found in the GO database in relation with the scope of this article.

Spot no. 1, whose expression levels were decreased (by 1.93-fold) in the retina of MPTP-treated monkeys compared to control animals (Table 1; Figure 2), was identified as α-enolase, encoded by the *ENO1* gene. This is a ubiquitous protein in adult tissues which participates in a variety of biological processes, such as growth control, hypoxia tolerance and allergy, but whose main function is to act as a glycolytic enzyme (Table 3) catalyzing the conversion of 2-phospho-D-glycerate into phosphoenolpyruvate.

Spot no. 2 (which exhibited a 2.97-fold reduction of staining intensity in the parkinsonian retina) was found to correspond to the β subunit of ATP synthase F_1_ component, encoded by the *ATP5B* gene. This is a constituent protein of mitochondrial complex V, i.e. the terminal complex in the electron transport chain, whose well known function is to carry out ATP synthesis from ADP by using the proton-motive force generated across the mitochondrial inner membrane.

Adjacent spots numbered 3 and 115 (see Figure 1C) were ascribed to presumable isoelectric variants of S-arrestin (showing content decreases of 2.82- and 2.17-fold, respectively). This is the product of the *SAG* gene, constituting a major component of the outer segments of rod photoreceptors where it participates in desensitization of the phototransduction cascade [36]. This is exerted through S-arrestin ability to bind and inhibit phosphorylated, light-activated rhodopsin, thereby preventing activation of cGMP phosphodiesterase mediated by transducin [36].

Spot no. 5 (whose normalized volume was increased by 2.52-fold in the parkinsonian retina) corresponded to β–crystallin B2, the product of *CRYBB2*. Crystallins, the majoritary structural components of the eye lens, are a superfamily of oligomer-forming proteins whose function is to increase the refractive index of the lens while maintaining its transparency. Yet, an additional role of these proteins in retinal neuron survival is becoming increasingly clear [37,38].

Spot no. 7 (displaying a 2.47-fold reduction in the MPTP-treated retina) was identified as the heat shock 70 kDa protein 8 (HSC70), also called heat shock cognate 71 kDa protein. This is the product of the *HSPA8* gene, which codes for a constitutively-expressed, cytosolic chaperone belonging to the Hsp70 family responsible for the correct folding and assembly of nascent polypeptides and degradation of misfolded proteins by chaperone-mediated autophagy [37].

Spot 12 was ascribed to stathmin, the product of the *STMN1* gene (presenting at lower levels, i.e. a 1.61-fold reduction, in treated monkeys), which is involved in cytoskeleton dynamics by preventing the assembly and promoting the disassembly of microtubules in a phosphorylation-regulated manner [39].

Spots 13 and 18 (showing a decrease in their normalized volumes by 2.10- and 1.99-fold, respectively) corresponded to the product of the *DDAH1* gene, N^G^, N^G^-dimethylarginine dimethylaminohydrolase-1. This enzyme hydrolyzes both asymmetric N^G^, N^G^-dimethyl-L-arginine (ADMA) and N^G^-monomethyl-L-arginine (MMA), two competitive inhibitors of all isoforms of nitric oxide synthase (NOS), to yield dimethylamine and L-citrulline, which no longer inhibit NOS [40]. The DDAH1 enzyme thus represents a mechanism for modulation of NO synthesis in both physiological and pathological states.

Spots 20 and 237 were also underexpressed in the retina of parkinsonian macaques (by 1.95- and 1.51-fold respectively) and were identified as pertaining to γ-enolase, a neuron-specific protein encoded by *ENO2*. Like the α isoenzyme above, γ-enolase participates in glycolysis by catalyzing the conversion of 2-phospho-D-glycerate into phosphoenolpyruvate. However, it also has neurotrophic and neuroprotective properties on neurons, promoting their survival in culture [41].

Spot 21 (whose intensity was found diminished 1.94-fold in the treated monkey retina) corresponded to the 78 kDa glucose-regulated protein (GRP78 or BiP), the product of the *HSPA5* gene. This is a chaperone mainly located at the endoplasmic reticulum (ER) that, alike Hsc70, belongs to the Hsp70 protein family. GRP78 not only is involved in correct protein folding and the assembly of protein complexes in the ER lumen, but also exhibits anti-apoptotic functions in neurons [42].

MS analysis allowed the identification of spot 31 (reduced by 1.37 fold in MPTP monkeys) as the *GAPDH* gene-encoded glyceraldehyde 3-phosphate dehydrogenase. This is a NAD^+^-dependent, relevant enzyme in the glycolytic pathway that catalyzes the phosphorylation-coupled oxidation of D-glyceraldehyde 3-phosphate to yield 1,3-bisphosphoglycerate. However, GAPDH has roles additional to its classical function as a glycolytic enzyme, also acting as a mediator of apoptosis promoted by cytotoxic stressors in neurons [43] and bearing a modulatory function of microtubule organization and dynamics [44].

Spot 33 was found to represent inorganic pyrophosphatase (showing a 1.67-fold in its normalized volume in the retina of parkinsonian vs. control individuals), coded for by the *PPA1* gene. This is a relevant enzyme in phosphate metabolism carrying out the hydrolysis of pyrophosphate (PPi) to yield two inorganic phosphate (Pi) molecules.

Spot 34 (whose intensity in the MPTP-treated retina was 1.65-fold lower than normal) was identified as calbindin D28k, the product of *CALB1*. This is a ubiquitous, Ca^2+^-binding protein with a relevant role in modulation of cytosolic Ca^2+^ levels and a proposed neuroprotective role [45,46].

The second protein found overexpressed in parkinsonian individuals (by 1.41-fold) was that represented by spot 37, which was ascribed by MS analysis to nucleoside diphosphate kinase B (NDPK B), encoded by the *NME2* gene. This is a cytoplasmic enzyme involved in the synthesis of nucleoside triphosphates, catalyzing the conversion of XTP plus YDP into XDP plus YTP (where X and Y are nucleosides other than adenosine) through formation of a His-phosphorylated enzyme intermediary form, although it may also act as a transcriptional activator [47]. In the retina it provides a key role in recycling GTP for phototransduction [48].

Spot 42 (which exhibited a volume reduction by 1.56-fold in the MPTP-treated retina) was found to correspond to the heme-containing, 5A subunit of cytochrome c oxidase (COX), encoded by *COX5A*. This is a constituent protein of mitochondrial complex IV, i.e. the terminal enzyme of the electron respiratory chain whose well known function is the transfer of electrons from cytochrome c to O_2_.

Finally, spot 91 was identified as γ-synuclein, encoded by the *SNCG* gene (and whose levels turned out to be 1.56-fold underexpressed in the treated monkeys). This protein plays a role in neurofilament network integrity and is thought to modulate axonal architecture during development and in the adult stage [49,50].

### Validation of Differentially-Expressed Proteins

**Figure 3:**
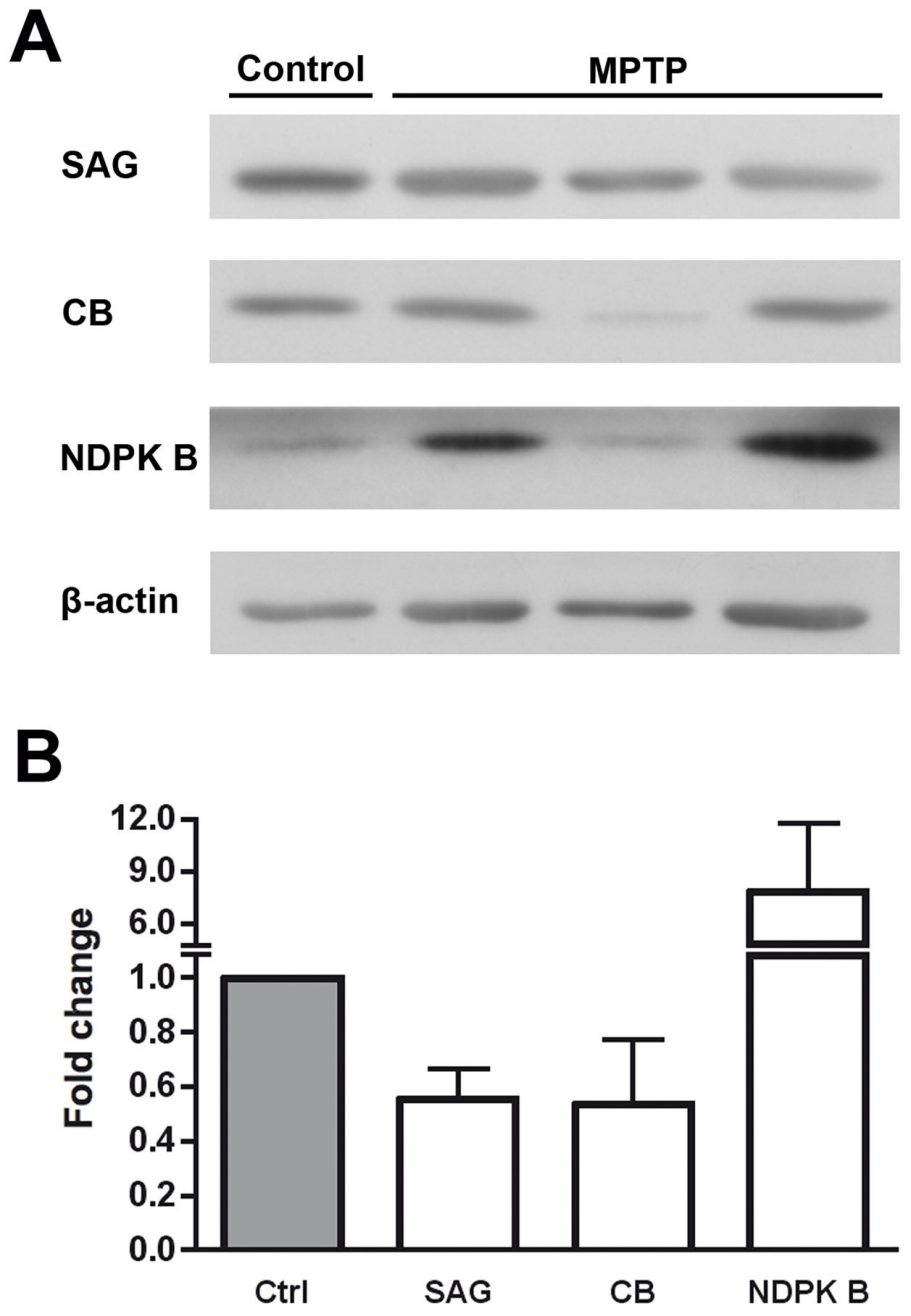
Western blotting validation of differentially expressed proteins in the retina of parkinsonian monkeys. **A**. Detection of S-arrestin (SAG; 48 kDa), calbindin (CB; 28 kDa) and nucleoside diphosphate kinase B (NDPK B; 17 kDa) in MPTP-treated and untreated monkeys by Western blotting. β-Actin (42 kDa) levels are shown as loading controls. **B**. The amount of protein present in each band was densitometrically measured and the value obtained for each detected protein was normalized to β-actin levels. Protein expression fold changes are shown, obtained as the ratio between MPTP-treated and control values, and taking the latter as 1.0. Bars represent the average ± SEM (n = 3).

Once analyzed and identified a series of proteins that showed differential expression levels between parkinsonian monkeys treated with the neurotoxin MPTP and controls, we proceeded to validate the reliability of our proteomic approach by Western blotting. With this purpose, three proteins were selected for the sake of being involved in phototransduction (S-arrestin and NDPK B) or being a good marker for cone photoreceptors in primates (calbindin) [51], and their levels detected in retinal extracts from parkinsonian and control monkeys are shown in Figure 3A. The expression fold changes shown in Figure 3B were calculated as the ratio between the MPTP-treated and control normalized values. We confirmed that levels of S-arrestin and calbindin were reduced, to 55.4% and 53.4% with respect to controls, respectively, which was coherent with decreases determined by DIGE. Also in keeping with the latter, NDPK B levels were found increased in the PD retina by Western blotting, by 7.84 fold as compared to untreated subjects. These experiments were indicative that DIGE combined with MS yielded results in the same direction than immunoblotting regarding differential expression of proteins in the retina of MPTP-treated monkeys.

## Discussion

Proteomics approaches have been previously used to address molecular alterations associated with a number of neurodegenerative disorders, including PD [6,14,52–55], Alzheimer disease (AD) [56–58] and Down syndrome [56], as well as those accompanying aging [59], in the brains of both human patients and animal models. Proteomic comparative analyses have also been undertaken on the retinas of human patients and animal models of Leber congenital amaurosis [60], age-related macular degeneration (AMD) [61–63], glaucoma [64] and diabetic retinopathy (DR) [65–68]. However, research at the proteome level of molecular alterations associated with PD in the retina of human or parkinsonian experimental animals has not been conducted so far. We have undertaken in this work a proteomic analysis of molecular alterations present in the retina of monkeys treated with the proneurotoxin MPTP, an animal model of parkinsonism where our group has previously described the existence of significant retinal impairments at the morphological and synaptic levels [21]. Proteins found in this work to undergo a decrease (most cases) or increase in their abundance in this animal model can be grouped into three main functional categories, as follows.

### Enzymes Involved in Energy Metabolism

We have determined in the retina of parkinsonian monkeys a lower abundance of enzymes involved in glycolysis, such as GAPDH and α- and γ-enolases. A reduced content of GAPDH has also been reported in the retina of streptozotocin-treated, DR rats [65]. Regarding enolases, the α polypeptide is expressed in most tissues, while the γ subunit is present exclusively in neurons, where functional enolase dimers mainly consist of γ/γ homodimers and α/γ heterodimers [69,70]. γ-Enolase plays roles additional to its function as a glycolytic enzyme, exhibiting neurotrophic and neuroprotective effects on cultured neocortical, mesencephalic and spinal cord neurons [41,71]. Alterations in γ-enolase levels have been reported in the cerebral cortex of mice with a KO mutation in the *Park2* gene, encoding parkin [55]. Here it is relevant to emphasize that loss-of-function mutations in the human *PARK2* gene are associated with autosomal recessive PD with juvenile onset [72]. Regarding the retina, α-enolase is found underexpressed at the onset of human AMD [61], and in aged rats this enzyme is irreversibly inactivated by the covalent addition of 4-hydroxynonenal (4HNE) [73], a reactive aldehyde derived from polyunsaturated fatty acid peroxidation that also accumulates in the brain of *Park2* KO mice [14]. Both α- and γ-enolases undergo nitrosative modification as well in the AD human brain [74]. Therefore, oxidation or nitrosylation of these enzymes in the CNS could bring about an impairment in glycolysis-coupled energy production, with especially harming effects in retinal neurons given their high energetic demand. Our observation of a decrease in levels of GAPDH together with those of α- and γ-enolases suggests the operation in the retina of a feed-back negative regulatory mechanism of glycolysis in response to blockade of mitochondrial complex I. Other glycolytic enzymes downregulated in the pathological retina are aldolase C at the onset of AMD [61] and triose phosphate isomerase in alloxan-treated, DR model rats [67], while underexpressed Krebs cycle enzymes include pyruvate dehydrogenase in the midbrain of *Park2* KO mice [14] and rotenone-treated rats [75]. In keeping with our hypotesis, it has been previously proposed that neurons respond to induced inhibition of mitochondrial respiration and increasing of reactive oxygen species (ROS) by actively downregulating glycolysis (through the action of the ubiquitin-proteasome pathway), giving priority to the consumption of glucose to maintain an antioxidant status over its use to fulfill their bioenergetic requirements [76].

We have also observed a reduction in the content of the 5A subunit of COX and β subunit of ATP synthase in the retina of parkinsonian monkeys, two enzymes that are well known constituents of mitochondrial complexes IV and V, respectively. A reduced activity or presence of these or other mitochondrial respiratory chain components has been reported in the brains of idiopathic PD, AD and Down syndrome cases [56,77–79], which has been correlated with a reduced respiratory capacity of mitochondria from *Park2* KO mice [14]. In the same context, the contents of both α and β subunits of ATP synthase F_1_ complex are decreased in the retinal pigment epithelium of AMD patients [63], as it is the case for the d subunit of the F_0_ complex (H^+^ channel) in the neural retina of DR rats [67]. Taken together, these data underscore that MPTP-induced mitochondrial complex I dysfunction can lead to alterations in the levels of other components of the respiratory chain, in analogy to what occurs in PD [6,75,80] and other neurodegenerative diseases affecting the brain and/or the retina. In a related fashion, an additional enzyme whose levels we have found decreased in the parkinsonian monkey retina is inorganic pyrophosphatase 1 (PPA1), which is responsible for modulating Pi levels and is intimately linked to cell survival [81,82]. Given that its activity is coupled to reactions in which PPi is released, and that it provides a thermodynamic pull for many biosynthetic reactions, its lower levels found in our experimental system are consistent with a situation in which the production of ATP is decreased due to the MPTP-provoked mitochondrial dysfunction.

### Neuroprotective Mechanisms

We have detected alterations in the expression of a series of proteins directly or indirectly related to neuronal protection against a variety of stresses, either endogenous or exogenous, in the retina of MPTP-treated monkeys. First, we have found underexpressed two members of the 70 kDa family of heat shock proteins (HSP70), namely HSC70 and GRP78, compared to control individuals. HSC70 is a molecular chaperone that functions to maintain protein homeostasis in cells under normal and stress conditions [37]. Its pattern of distribution in the rat retina includes all retinal layers, except photoreceptor outer segments [83], and its expression levels have been found by proteomic analysis diminished in the striatum and cortex of *Park2* KO mice [55]. As well, a significant mRNA underexpression of its encoding gene, *HSPA8*, occurs with age in the primate retina [84]. It follows that its ameliorated levels found in PD or aging could underlie the neurodegeneration occurring in these conditions, as proposed for both the brain [58] and the retina [84]. On the other hand, the intravitreal injection of HSC70 is protective against acute light-induced damage in rats, increasing the number of surviving photoreceptors [85]. Relatedly, GRP78 is a rough-ER chaperone working in the unfolded-protein response to prevent ER stress-induced cell death in the brain [86,87] and retinal [88] neurons, a phenomenon associated with a number of neurodegenerative diseases [37] including retinal disorders [59,89]. GRP78 is expressed in photoreceptors, including their outer segments [90,91], and attenuated levels of this protein have been documented in the brain of PD patients [86] and the cortex of *Park2* KO mice [55], this pointing out to the dysfunction of a neuroprotective mechanism additional to HSC70 in our MPTP-treated monkeys.

We have detected DDAH1 levels to be lower in the retina of PD macaques in this work, while DDAH2 has been found downregulated in the DR rat retina [67]. These are monomeric hydrolases expressed in the brain and other organs able to degrade ADMA and MMA, two methylated L-arginine derivatives which act as endogenous inhibitors of NOS, thereby contributing to regulated NO levels in numerous disease states [40]. Hence, a relationship exists between NO and protein misfolding in neurodegenerative disorders [92] such as PD, where nitrosative stress very often accompanies oxidative stress [6,12,93]. In this context, levels of proteins protective against the latter are diminished in the brains of PD patients and *Park2* KO mice, together with a reduced antioxidant capacity and ability to respond to ROS generation [14,52]. In a related fashion, parkin is found S-nitrosylated in the brains of parkinsonian animal models and patients, this resulting in inhibition of its ubiquitin E3 ligase activity and neuroprotective function [93–95]. As well, TH itself is known to undergo Tyr nitration in MPTP-treated mice, also resulting in its inactivation [96]. Therefore, the decreased DDAH1 levels in the retina of our parkinsonian monkeys should result in lower NO levels, and thus be oriented at counteracting MPTP-derived nitrosative stress consequences, in the same line of thought as proposed for dopaminergic neurons of baboons [97] and mice [98,99] brains, and for DDAH2 downregulation in the DR rat retina [67].

As a difference with other proteins above, β-crystallin B2 was found overexpressed in the retina of parkinsonian monkeys. α-, β- and γ-crystallins, in addition to being the main structural constituents of the lens, are present in the neural retina and brain, and their additional role as oxidative stress-inducible chaperones of the small HSP family is especially clear for α-crystallins [38,100]. The localization of β-crystallin B2 in the mouse retina includes the inner segments of photoreceptors, some cells of the inner nuclear layer and ganglion cells [101]. Overexpression of β-crystallin B2 has also been reported in the retinas of rats suffering DR [68,102], optic nerve physical damage [103] or normal aging [73]. Other α-, β- and/or γ-crystallins also become upregulated in the retina of several genetic rodent models of retinitis pigmentosa [104,105] and DR model rats [66–68,102], and under light-induced retinal degeneration [105], as well as in the human AD brain [57]. All these investigations have led to the view that crystallins could act, not only as chaperones, but also as factors promoting the regrowth of neuronal processes, thereby participating in axonal regeneration upon tissue damage [14,37,103]. Even, their level increases (especially of α-crystallins) have been correlated with the rate of photoreceptor loss [105].

Stathmin, encoded by the *STMN1* gene, belongs to a family of microtubule-destabilizing proteins with an important role in cytoskeleton reorganization taking place during neuronal differentiation in development and after lesion in adulthood [39,106]. It is thus involved in modulating axon integrity in the nervous system, in the context of retinal plasticity and in regeneration processes. The levels of this protein appear reduced in the brains of patients with AD [107] and Down syndrome [108], as well as in the AMD retinal proteome [61]. Stathmin decrease in the PD monkey retina could either reflect the failure of a system in charge of maintaining microtubule stability and axonal integrity in the retina, such dysfunction contributing to neuronal degeneration. On the other hand, since stathmin is a microtubule-depolymerizing protein, its reduction could be aimed at counteracting neuronal morphology derangement, as it could also be the case for GAPDH [44]. Further research is needed to discern between these hypotheses.

Calbindin is a multifunctional cytoplasmic protein crucial in the modulation of cytosolic Ca^2+^ levels and intracellular signalling. It has been found that dopaminergic neurons that express calbindin in the substantia nigra are spared from degeneration in PD patients and MPTP-treated monkeys and mice [45], and levels of this protein are reduced in the brain of old mice [59]. This indicates that dysregulation of Ca^2+^ levels could be one of the mechanisms underlying brain and retinal neurodegeneration associated with parkinsonism, and points out a likely preventive role of calbindin in this context. In a related fashion, the levels of another potentially-neuroprotective Ca^2+^-binding protein, the neuron-specific calretinin, are diminished in the cortex of *Park2* KO mice [55] and in the retina of AMD patients [61].

Last in this group of neuroprotective proteins, γ-synuclein was also found underexpressed in the retina in our PD monkey model. This is a cytoplasmic protein whose function in the retina is poorly understood, and that is mainly contained in some ganglion cells and in optic nerve fibres [109]. It has been proposed to play a role as a retinal chaperone, working to restore the correct folding of anomalous proteins and prevent their aggregation [110]. In this context, γ-synuclein has shown some neuroprotective effects on a photoreceptor cell line expressing a retinitis pigmentosa-causative dominant allele of the rhodopsin gene, P23H [111], encoding a misfolded, cytotoxic variant of this protein.

### Visual Signal Transduction

S-Arrestin, also known as rod arrestin, is one of the most abundant proteins in the outer segments, where it participates in the regeneration phase of the phototransduction cascade with restoration of high cGMP levels [36]. It binds to photoactivated, phosphorylated rhodopsin, thereby preventing its interaction with transducin and ensuing activation of cGMP phosphodiesterase 6B (PDE6B) [48]. Therefore, the attenuated S-arrestin expression we have found in MPTP monkeys would act in the sense of keeping PDE6B active and, consequently, Na^+^ and Ca^2+^ cGMP-gated channels closed, with ensuing cell hyperpolarization and cessation of Glu release in photoreceptor axon terminals. Somewhat surprisingly, interaction of S-arrestin with α-enolase has been recently reported to modulate the activity of the latter, in the sense of reducing its catalytic rate [112]. Conversely, and more in line with our results, mutations in the *SAG* gene have been associated with Oguchi disease, a variant of retinitis pigmentosa [113].

We have also found that NDPK B is overexpressed in the retina of parkinsonian monkeys. This protein, among other functions, provides the necessary intracellular GTP for transducin and guanylate cyclase activities. Two hexameric isoforms of this enzyme, A and B, are present in the retina [48], of which the B isoform is known to form a complex with the Gβγ heterodimer of various G proteins, including transducin. Actually, NDPK B and transducin copurify from rod outer segment membranes [114], and transducin is long known to mediate interaction of NDPK (A and B) with (bleached) rod outer segment membranes [115]. Furthermore, transducin Gβγ is likely to be a substrate for His phosphorylation by NDPK B in the retina [116]. Our results thus suggest that in parkinsonian monkeys an increased activation of transducin could take place independently of photoactivated rhodopsin in an NDPK B-mediated fashion, this enhancing PDE6B activity and thereby decreasing cGMP intracellular levels. The NDPK B increase would thus act in the same direction as S-arrestin underexpression, i.e. contributing to keep Na^+^ and Ca^2+^ cGMP-gated channels closed. Very interestingly, the NDPK B-encoding mRNA is overexpressed in the degenerating retina of the *rd/rd* mouse, a retinitis pigmentosa model lacking PDE6B [104].

The alteration in S-arrestin and NDPK B levels reflects, in our belief, a protective response by photoreceptors to mitochondrial damage. Since NDPK B appears to be a stress-inducible protein, its rise together with α- and β-crystallin upregulation may be a component of the stress response to photoreceptor loss and altered retinal architecture [104]. In this picture, Na^+^/Ca^2+^ channel closure would be aimed at preventing a sustained elevation of intracellular Ca^2+^, which would trigger apoptotic death of photoreceptors [117], as well as at avoiding the energy expenditure required to maintain the dark current [118]. In fact, such high energetic demand could not be fulfilled by mitochondria in a situation of depressed metabolism plus oxidative stress, where the additionally superimposed calbindin downregulation would hamper the buffering of such Ca^2+^ increase [119].

## Conclusions

This study is the first in providing direct evidence of molecular alterations in the retina associated with PD, obtained from a proteomics approach. Proteins found with altered levels can be classified as related to: i) impairment of energy metabolism (particularly concerning the glycolytic pathway and mitochondrial ATP synthesis); ii) neuronal protection against various stresses; and iii) visual signal transduction. Most of these alterations act in the sense of potentiating neuroprotective mechanisms aimed at counteracting MPTP oxidative damage and/or promoting cell survival. The downregulated levels found for proteins crucial for glycolysis and mitochondrial electron transport chain reflect, in our view and in consistency with previous proteomic studies on parkinsonian human and animal-model brains, a feed-back cellular response to mitochondrial complex I inactivation oriented to diminish the global rate of cellular energy metabolism and/or the excess production of ROS ensuing mitochondrial dysfunction. Other alterations occurred in the sense of ameliorating nitrosative stress, increasing chaperone activity, counteracting morphological degeneration or lowering the phototransduction rate itself. Nevertheless, some decreases of protein expression (such as the lower presence of HSC70, GRP78, calbindin and γ-synuclein, and as discussed above for stathmin) may simply reflect a failure or dysregulation of protective neuronal systems. This would be a direct consequence of the intracellularly generated oxidative stress and subsequent intracellular damage, similar to that seen in the aging process [84], in cells that have irreversibly entered the apoptotic way and thereby contributing to its progression. This is consistent with the finding that in the human PD substantia nigra alterations in the mRNA levels of some genes do accelerate cell death activity, whereas other retard or attempt to compensate this process [120], as also concluded from other proteomics studies tackling neurodegenerative diseases. It follows that a complex set of mechanisms including neuroprotective responses as well as neuronal death appears to be involved in PD onset or progression, whose interplay should require a good number of further studies to be clarified.

Loss of dopaminergic amacrines and their postsynaptic cells in the peripheral retina of MPTP-treated monkeys has been suggested to impair the scotopic visual pathway [21]. However, recent studies on PD patients indicate that foveal vision is impaired in humans, as evidenced by thinning of the inner retina in the macular region detected by optical coherence tomography (OCT) [15,17,121], which together with loss of electrical activity recorded in the fovea reflects a deficit in the photopic pathway [122–124]. Differences in the retinal proteomes of monkeys treated with the parkinsonism inducer MPTP compared to control animals point out to important functional groupings (energy metabolism, protein folding vs. degradation, stress response, neuronal survival vs. apoptosis, signal transduction pathways, cytoskeletal dynamics and others) and particular protein actors that have been implicated as well in the pathogenesis of a number of CNS disorders in humans and animal models, some of which also affect the retina. It can thus be inferred that common intracellular agents and molecular mechanisms, whose involvement in neurodegenerative processes in the brain is increasingly known, are implicated as well in retinal degenerations, including PD-derived derangement of retinal neurons and retina-specific diseases. Further studies are needed to more exactly dilucidate the role of each of them in retinal neurodegeneration in monkeys treated with MPTP, and in terms of PD in general, and to unravel the levels at which they are interrelated. This information could eventually be useful to develop pharmacotherapies targeting fundamental biochemical defects aimed at retarding disease progression and/or alleviating neurodegeneration symptoms in the the retina and brain.

## Supporting Information

Figure S1 MS/MS spectrum of a peptide identified by *de novo* sequencing. The fragmentation spectrum of a calbindin precursor peptide (spot no. 34) is shown, which was identified by *de novo* sequencing using the Sherenga algorithm. Its amino acid sequence obtained from database search using the Spectrum Mill software is indicated at the top of the image (MSTag), and that interpreted by the Sherenga algorithm is given below (upper and lower graphs, respectively). Graphs show the relative intensity (%) of each peptide fragment plotted as a function of its m*/z* value, indicated above its corresponding peak.
(TIF)

Table S1 Peptides identified by MALDI-TOF MS. (DOCX)

Table S2 Peptides identified by LC-MS/MS. (DOCX)
