# Supplementary figures and images for: Alterations in Energy Metabolism, Neuroprotection and Visual Signal Transduction in the Retina of Parkinsonian, MPTP-Treated Monkeys
Source: PLoS One. 2013 Sep 5;8(9):e74439. doi: 10.1371/journal.pone.0074439 (PMC3764107; doi:10.1371/journal.pone.0074439)

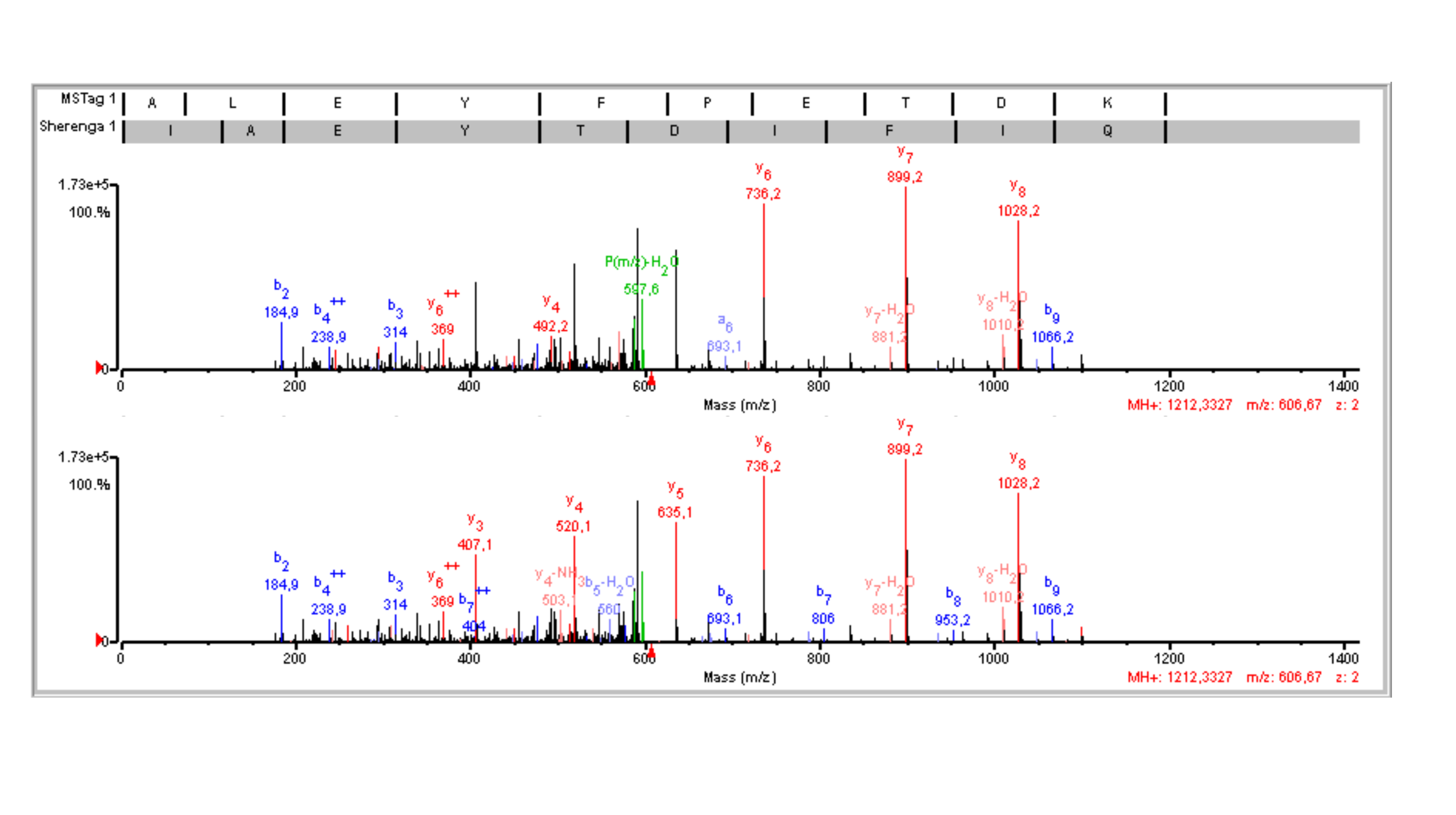

Supplement: Figure S1 — The fragmentation spectrum of a calbindin precursor peptide (spot no. 34) is shown, which was identified by de novo sequencing using the Sherenga algorithm. Its amino acid sequence obtained from database search using the Spectrum Mill software is indicated at the top of the image (MSTag), and that interpreted by the Sherenga algorithm is given below (upper and lower graphs, respectively). Graphs show the relative intensity (%) of each peptide fragment plotted as a function of its m/z value, indicated above its corresponding peak. (TIF) [file pone.0074439.s001.tif]
