# Supplementary material for: Alterations in Energy Metabolism, Neuroprotection and Visual Signal Transduction in the Retina of Parkinsonian, MPTP-Treated Monkeys
Source: PLoS One. 2013 Sep 5;8(9):e74439. doi: 10.1371/journal.pone.0074439 (PMC3764107; doi:10.1371/journal.pone.0074439)
Supplement: Table S1 — (DOCX) [file pone.0074439.s002.docx]

**Table S1.** Peptides identified by MALDI-TOF MS.

| **Spot no.^a^** | **Protein^a^** | **Number of peptides^b^** | **Identified peptides^c^** |
| --- | --- | --- | --- |
| 1 | α-Enolase | 16/46 | (R)AAVPSGASTGIYEALELR(D) |
|  |  |  | (K)FGANAILGVSLAVCK(A) |
|  |  |  | (R)HIADLAGNSEVILPVPAFNVINGGSHAGNK(L) |
|  |  |  | (K)LAMQEFMILPVGAANFR(E) |
|  |  |  | (K)LAmQEFMILPVGAANFR(E) |
|  |  |  | (K)LAmQEFmILPVGAANFR(E) |
|  |  |  | (R)IGAEVYHNLKNVIK(E) |
|  |  |  | (K)DATNVGDEGGFAPNILENK(E) |
|  |  |  | (K)VVIGMDVAASEFFR(S) |
|  |  |  | (K)VVIGMDVAASEFFR(S) |
|  |  |  | (R)YISPDQLADLYK(S) |
|  |  |  | (K)DYPVVSIEDPFDQDDWGAWQK(F) |
|  |  |  | (K)FTASAGIQVVGDDLTVTNPK(R) |
|  |  |  | (K)VNQIGSVTESLQACK(L) |
|  |  |  | (K)LAQANGWGVMVSHR(S) |
|  |  |  | (R)SGETEDTFIADLVVGLCTGQIK(T) |
| 5 | β-Crystallin B2 | 15/20 | (K)AGSVLVQAGPWVGYEQANCK(G) |
|  |  |  | (K)GEQFVFEK(G) |
|  |  |  | (K)GEYPRWDSWTSSR(R) |
|  |  |  | (R)WDSWTSSR(R) |
|  |  |  | (K)IILYENPNFTGK(K) |
|  |  |  | (K)IILYENPNFTGKK(M) |
|  |  |  | (K)KMEIIDDDVPSFHAHGYQEK(V) |
|  |  |  | (K)MEIIDDDVPSFHAHGYQEK(V) |
|  |  |  | (R)VQSGTWVGYQYPGYR(G) |
|  |  |  | (R)GLQYLLEK(G) |
|  |  |  | (K)GDYKESSDFGAPHPQVQSVR(R) |
|  |  |  | (K)ESSDFGAPHPQVQSVR(R) |
|  |  |  | (R)IRDMQWHQR(G) |
|  |  |  | (R)IRDmQWHQR(G) |
|  |  |  | (R)DMQWHQR(G) |
| 18 | N^G^,N^G^-Dimethylarginine dimethylamino-hydrolase 1 (DDAH1) | 16/44 | (R)ALPESLGQHALR(S) |
|  |  |  | (K)GEEVDVAR(A) |
|  |  |  | (R)QHQLYVGVLGSK(L) |
|  |  |  | (K)LQLNIVEMK(D) |
|  |  |  | (K)LQLNIVEMKDENATLDGGDVLFTGR(E) |
|  |  |  | (K)DENATLDGGDVLFTGR(E) |
|  |  |  | (R)EFFVGLSK(R) |
|  |  |  | (R)GAEILADTFK(D) |
|  |  |  | (K)DYAVSTVPVADGLHLK(S) |
|  |  |  | (K)SFCSMAGPNLIAIGSSESAQK(A) |
|  |  |  | (K)IMQQMSDHR(Y) |
|  |  |  | (K)LTVPDDIAANCIYLNIPNK(G) |
|  |  |  | (R)TPEEYPESAK(V) |
|  |  |  | (K)LKDHMLIPVSMSELEK(V) |
|  |  |  | (K)LKDHmLIPVSMSELEK(V) |
|  |  |  | (K)VDGLLTCCSVLINK(K) |
| 33 | Inorganic pyrophosphatase (PPA1) | 18/40 | (–)mYSWGVLAVRR(R) |
|  |  |  | (R)AAPFSLEYR(V) |
|  |  |  | (K)GQYISPFHDIPIYADK(D) |
|  |  |  | (K)GQYISPFHDIPIYADKDVFHMVVEVPR(W) |
|  |  |  | (K)DVFHMVVEVPR(W) |
|  |  |  | (R)YVANLFPYK(G) |
|  |  |  | (K)GYIWNYGAIPQTWEDPGHNDK(H) |
|  |  |  | (K)HTGCCGDNDPIDVCEIGSK(V) |
|  |  |  | (K)VLGILAMIDEGETDWK(V) |
|  |  |  | (K)VIAINVDDPDAANYNDINDVK(R) |
|  |  |  | (K)VIAINVDDPDAANYNDINDVKR(L) |
|  |  |  | (R)LKPGYLEATVDWFR(R) |
|  |  |  | (R)YKVPDGKPENEFAFNAEFK(D) |
|  |  |  | (K)VPDGKPENEFAFNAEFK(D) |
|  |  |  | (K)DKDFAIDIIK(S) |
|  |  |  | (K)GISCMNTTVSESPFK(C) |
|  |  |  | (K)GISCmNTTVSESPFK(C) |
|  |  |  | (R)AIVDALPPPCESACTVPTDVDK(W) |
| 237 | γ-Enolase | 21/44 | (R)GNPTVEVDLYTAK(G) |
|  |  |  | (R)AAVPSGASTGIYEALELR(D) |
|  |  |  | (K)AVDHINSTIAPALISSGLSVVEQEK(L) |
|  |  |  | (K)LDNLMLELDGTENK(S) |
|  |  |  | (K)FGANAILGVSLAVCK(A) |
|  |  |  | (R)HIAQLAGNSDLILPVPAFNVINGGSHAGNK(L) |
|  |  |  | (K)LAMQEFMILPVGAESFR(D) |
|  |  |  | (K)LAmQEFMILPVGAESFR(D) |
|  |  |  | (R)LGAEVYHTLK(G) |
|  |  |  | (K)DATNVGDEGGFAPNILENSEALELVK(E) |
|  |  |  | (K)IVIGMDVAASEFHR(D) |
|  |  |  | (K)IVIGmDVAASEFHR(D) |
|  |  |  | (R)DGKYDLDFK(S) |
|  |  |  | (R)YITGDQLGTLYQDFVR(D) |
|  |  |  | (R)DYPVVSIEDPFDQDDWAAWSK(F) |
|  |  |  | (K)FTANVGIQIVGDDLTVTNPK(R) |
|  |  |  | (K)VNQIGSVTEAIQACK(L) |
|  |  |  | (K)LAQENGWGVMVSHR(S) |
|  |  |  | (K)LAQENGWGVmVSHR(S) |
|  |  |  | (R)SGETEDTFIADLVVGLCTGQIK(T) |
|  |  |  | (R)IEEELGDEAR(F) |

**^a^**The spot number and the corresponding protein identified in each case are indicated.

**^b^**The number of peptides leading to each protein identification (selected on the basis of their *m/z* values matching those of peptide segments of the identified protein) is indicated together with the total number of peptides obtained by MALDI-TOF and used for database screening (separated by a slash).

**^c^**The sequence of the different peptides whose calculated *m/z* corresponds to that obtained by MALDI-TOF is shown. Residues in parentheses at both ends of each peptide were inferred from tryptic digestion, except (−), unknown. Lowercase m stands for oxidized Met.
