# Supplementary material for: Alterations in Energy Metabolism, Neuroprotection and Visual Signal Transduction in the Retina of Parkinsonian, MPTP-Treated Monkeys
Source: PLoS One. 2013 Sep 5;8(9):e74439. doi: 10.1371/journal.pone.0074439 (PMC3764107; doi:10.1371/journal.pone.0074439)
Supplement: Table S2 — (DOCX) [file pone.0074439.s003.docx]

**Table S2.** Peptides identified by LC-MS/MS.

| **Spot no.** | **Protein** | **Identified peptides** | **Score** | **z** | **SPI (%)** | **MH^+^ mass (Da)** |
| --- | --- | --- | --- | --- | --- | --- |
| 1 | α-Enolase | (K)VNQIGSVTESLQACK(L) | 24.97 | 2 | 96.4 | 1633.8 |
|  |  | (R)AAVPSGASTGIYEALELR(D) | 24.00 | 2 | 94.9 | 1804.9 |
|  |  | (R)SGKYDLDFKSPDDPSR(Y) | 23.97 | 3 | 96.2 | 1826.9 |
|  |  | (K)VVIGmDVAASEFFR(S) | 23.31 | 2 | 95.5 | 1540.8 |
|  |  | (K)LAmQEFmILPVGAANFR(E) | 23.09 | 3 | 95.8 | 1908.0 |
|  |  | (K)DATNVGDEGGFAPNILENK(E) | 22.26 | 2 | 97.1 | 1960.9 |
|  |  | (R)YISPDQLADLYK(S) | 19.78 | 2 | 96.6 | 1425.7 |
|  |  | (R)GNPTVEVDLFTSK(G) | 19.24 | 2 | 91.4 | 1406.7 |
|  |  | (K)LmIEmDGTENK(S) | 18.10 | 2 | 100.0 | 1280.6 |
|  |  | (R)LGAEVYHTLK(G) | 16.40 | 2 | 87.0 | 1130.6 |
|  |  | (K)LAQANGWGVmVSHR(S) | 14.96 | 3 | 74.2 | 1525.8 |
|  |  | (K)KLNVTEQEKIDK(L) | 14.60 | 3 | 82.1 | 1444.8 |
| 2 | ATP synthase, subunit β | (R)TREGNDLYHEMIESGVINLK(D) | 23.00 | 3 | 100.0 | 2318.1 |
|  |  | (R)FTQAGSEVSALLGR(I) | 22.50 | 2 | 100.0 | 1435.8 |
|  |  | (K)VLDSGAPIKIPVGPETLGR(I) | 22.32 | 3 | 96.5 | 1919.1 |
|  |  | (K)VALVYGQMNEPPGAR(A) | 21.92 | 2 | 97.5 | 1601.8 |
|  |  | (K)TVLIMELINNVAK(A) | 21.50 | 2 | 100.0 | 1457.8 |
|  |  | (R)VALTGLTVAEYFR(D) | 20.42 | 2 | 98.7 | 1439.8 |
|  |  | (R)LVLEVAQHLGESTVR(T) | 20.06 | 2 | 97.3 | 1650.9 |
|  |  | (R)VALTGLTVAEYFRDQEGQDVLLFIDNIFR(F) | 20.00 | 3 | 96.3 | 3342.7 |
|  |  | (R)TIAMDGTEGLVR(G) | 19.22 | 2 | 99.1 | 1262.6 |
|  |  | (R)IPSAVGYQPTLATDMGTMQER(I) | 18.62 | 3 | 87.8 | 2266.1 |
|  |  | (R)EGNDLYHEMIESGVINLK(D) | 18.21 | 3 | 96.3 | 2061.0 |
|  |  | (R)IMDPNIVGSEHYDVAR(G) | 17.62 | 3 | 95.1 | 1815.9 |
|  |  | (K)AHGGYSVFAGVGER(T) | 16.93 | 3 | 91.6 | 1406.7 |
|  |  | (R)FLSQPFQVAEVFTGHMGK(L) | 16.59 | 3 | 93.7 | 2023.0 |
|  |  | (R)AIAELGIYPAVDPLDSTSR(I) | 15.91 | 3 | 81.2 | 1988.0 |
|  |  | (R)IVAVIGAVVDVQFDEGLPPILNALEVQGR(E) | 15.02 | 3 | 84.7 | 3031.7 |
|  |  | (R)IMNVIGEPIDER(G) | 14.73 | 2 | 92.3 | 1385.7 |
|  |  | (K)IPVGPETLGR(I) | 14.07 | 2 | 87.3 | 1038.6 |
|  |  | (K)VLDSGAPIK(I) | 13.68 | 2 | 96.5 | 899.5 |
|  |  | (R)IMNVIGEPIDERGPIK(T) | 13.36 | 3 | 71.1 | 1781.0 |
|  |  | (K)VVDLLAPYAK(G) | 12.52 | 2 | 85.3 | 1088.6 |
| 3 | S-Arrestin | (R)VQVYPPVGAASTPTK(L) | 15.08 | 2 | 92.4 | 1514.8 |
|  |  | (K)VYVTLTCAFR(Y) | 14.92 | 2 | 92.8 | 1229.6 |
|  |  | (K)LQESLLKK(L) | 14.52 | 3 | 93.2 | 958.6 |
|  |  | (R)DKSVTIYLGK(R) | 13.56 | 2 | 87.8 | 1123.6 |
|  |  | (K)KVYVTLTCAFR(Y) | 12.71 | 3 | 79.1 | 1357.7 |
|  |  | (K)SCGVDFEVK(A) | 10.01 | 2 | 65.1 | 1040.5 |
| 7 | Heat shock 70 kDa protein 8 (HSC70) | (K)STAGDTHLGGEDFDNR(M) | 18.11 | 2 | 89.6 | 1691.7 |
|  |  | (K)NQVAmNPTNTVFDAKR(L) | 17.81 | 3 | 87.0 | 1805.9 |
|  |  | (K)DAGTIAGLNVLR(I) | 16.96 | 2 | 82.5 | 1199.7 |
|  |  | (K)NSLESYAFNMK(A) | 12.96 | 2 | 74.7 | 1303.6 |
|  |  | (R)LSKEEIERmVnEAEK(Y) | 8.60 | 3 | 71.2 | 1804.9 |
| 12 | Stathmin | (R)ASGQAFELILSPR(S) | 16.38 | 2 | 91.7 | 1388.8 |
|  |  | (K)AIEENNNFSK(M) | 15.20 | 2 | 83.7 | 1165.5 |
| 13 | N^G^,N^G^-Dimethylarginine dimethylamino-hydrolase 1 (DDAH1) | (K)DENATLDGGDVLFTGR(E) | 23.55 | 2 | 97.0 | 1679.8 |
|  |  | (R)TPEEYPESAK(V) | 21.15 | 2 | 95.2 | 1150.5 |
|  |  | (K)VDGLLTCCSVLINK(K) | 19.88 | 2 | 95.7 | 1591.8 |
|  |  | (R)ALPESLGQHALR(S) | 18.80 | 2 | 91.9 | 1291.7 |
|  |  | (K)LQLNIVEmK(D) | 18.44 | 2 | 93.1 | 1087.6 |
|  |  | (R)GAEILADTFK(D) | 18.07 | 2 | 93.4 | 1064.6 |
|  |  | (R)QHQLYVGVLGSK(L) | 16.41 | 3 | 80.4 | 1328.7 |
|  |  | (K)DYAVSTVPVADGLHLK(S) | 16.11 | 2 | 82.5 | 1684.9 |
| 18 | DDAH1 | (K)DENATLDGGDVLFTGR(E) | 21.92 | 2 | 98.9 | 1679.8 |
|  |  | (K)VDGLLTCCSVLINK(K) | 20.77 | 2 | 96.4 | 1591.8 |
|  |  | (K)SFCSMAGPNLIAIGSSESAQK(A) | 19.57 | 3 | 93.6 | 2155.0 |
|  |  | (K)LTVPDDIAANCIYLNIPNK(G) | 18.72 | 3 | 95.0 | 2144.1 |
|  |  | (K)LQLNIVEMK(D) | 16.90 | 2 | 95.6 | 1087.6 |
|  |  | (R)ALPESLGQHALR(S) | 16.83 | 2 | 93.3 | 1291.7 |
|  |  | (R)GAEILADTFK(D) | 16.81 | 2 | 95.8 | 1064.6 |
|  |  | (R)GAEILADTFKDYAVSTVPVADGLHLK(S) | 16.75 | 3 | 89.7 | 2730.4 |
|  |  | (K)LKDHMLIPVSMSELEK(V) | 15.29 | 3 | 88.2 | 1870.0 |
|  |  | (K)DYAVSTVPVADGLHLK(S) | 14.90 | 2 | 86.0 | 1684.9 |
|  |  | (R)TPEEYPESAKVYEK(L) | 14.52 | 3 | 75.0 | 1669.8 |
|  |  | (R)EFFVGLSKR(T) | 13.72 | 2 | 82.3 | 1082.6 |
|  |  | (R)QHQLYVGVLGSK(L) | 11.97 | 2 | 84.9 | 1328.7 |
| 20 | γ-Enolase | (K)VNQIGSVTEAIQACK(L) | 23.87 | 2 | 95.8 | 1617.8 |
|  |  | IEEEIGDEAR | 165**^a^** |  |  |  |
| 21 | Glucose-regulated 78 kDa protein (GRP78) | (R)ITPSYVAFTPEGER(L) | 19.32 | 2 | 94.6 | 1566.8 |
|  |  | (K)VTHAVVTVPAYFNDAQR(Q) | 17.25 | 3 | 85.2 | 1888.0 |
|  |  | (K)SQIFSTASDNQPTVTIK(V) | 16.34 | 3 | 78.9 | 1836.9 |
|  |  | (R)IINEPTAAAIAYGLDKR(E) | 15.40 | 3 | 93.3 | 1816.0 |
| 31 | Glyceraldehyde 3-phosphate dehydrogenase (GAPDH) | (K)LISWYDNEFGYSNR(V) | 20.59 | 2 | 96.3 | 1763.8 |
|  |  | (R)VPTANVSVVDLTCR(L) | 16.87 | 2 | 91.0 | 1530.8 |
|  |  | (K)VGVNGFGR(I) | 15.84 | 2 | 87.9 | 805.4 |
| 33 | Inorganic pyrophosphatase (PPA1) | (K)HTGCCGDNDPIDVCEIGSK(V) | 22.94 | 3 | 95.0 | 2133.9 |
|  |  | (K)mEIATKDPLNPIK(Q) | 20.82 | 3 | 90.8 | 1469.8 |
|  |  | (K)GISCmNTTVSESPFK(C) | 20.14 | 2 | 96.7 | 1657.8 |
|  |  | (K)GISCmNTTVSESPFKCDPDAAR(A) | 17.90 | 3 | 87.9 | 2443.1 |
|  |  | (R)AAPFSLEYR(V) | 17.22 | 2 | 95.7 | 1053.5 |
|  |  | (K)VIAINVDDPDAANYNDINDVKR(L) | 16.25 | 3 | 91.5 | 2444.2 |
|  |  | (R)YVANLFPYK(G) | 14.55 | 2 | 89.0 | 1114.6 |
|  |  | (K)DPLNPIKQDVK(K) | 14.25 | 2 | 81.5 | 1266.7 |
| 34 | Calbindin | (R)LLPVQENFLIK(F) | 17.43 | 2 | 93.7 | 1313.8 |
|  |  | IAEYTDIFIQ | 154**^a^** |  |  |  |
| 37 | Nucleoside diphosphate kinase B (NDPK B) | (R)GDFCIQVGR(N) | 17.74 | 2 | 89.9 | 1051.5 |
|  |  | (R)TFIAIKPDGVQR(G) | 15.03 | 2 | 83.3 | 1344.8 |
|  |  | (R)LMLGETNPADSKPGTIR(G) | 11.72 | 3 | 83.4 | 1799.9 |
| 42 | Cytochrome c oxidase (COX), subunit 5A | (K)GINTLVTYDLVPEPK(I) | 21.95 | 2 | 94.6 | 1658.9 |
|  |  | (R)LNDFASTVR(I) | 14.45 | 2 | 82.6 | 1022.5 |
| 91 | γ-Synuclein | (K)TKEQANAVSEAVVSSVNTVAAK(T) | 20.44 | 3 | 96.6 | 2203.2 |
|  |  | (K)TVEEAENIAVTSGVVR(K) | 20.22 | 2 | 95.4 | 1673.9 |
|  |  | (K)EQANAVSEAVVSSVNTVAAK(T) | 18.48 | 2 | 95.5 | 1974.0 |
|  |  | (K)TVEEAENIAVTSGVVRK(E) | 17.08 | 3 | 93.5 | 1802.0 |
|  |  | (K)TKENVVHSVTSVAEK(T) | 10.50 | 3 | 75.1 | 1627.9 |
|  |  | (K)EGVmYVGTK(T) | 10.14 | 2 | 87.4 | 983.5 |
| 115 | S-Arrestin | (K)ESYQDANLVFEEFAR(H) | 22.19 | 3 | 95.4 | 1817.8 |
|  |  | (R)KVQHAPLEmGPQPR(A) | 21.82 | 3 | 96.0 | 1587.8 |
|  |  | (K)VYVTLTCAFR(Y) | 20.74 | 2 | 95.7 | 1229.6 |
|  |  | (R)VQVYPPVGAASTPTK(L) | 19.55 | 2 | 93.7 | 1514.8 |
|  |  | (R)LmHPQPEDPAK(E) | 18.04 | 2 | 85.3 | 1262.6 |
|  |  | (K)TLTLLPLLANNR(E) | 17.14 | 2 | 75.4 | 1338.8 |
|  |  | (K)VQHAPLEMGPQPR(A) | 16.15 | 3 | 83.3 | 1459.7 |
|  |  | (K)IKHEDTNLASSTIIK(E) | 14.25 | 3 | 86.4 | 1669.9 |
|  |  | (K)SCGVDFEVK(A) | 13.05 | 2 | 81.9 | 1040.5 |
|  |  | (R)DKSVTIYLGK(R) | 12.70 | 2 | 85.0 | 1123.6 |
|  |  | (K)SVTIYLGK(R) | 12.39 | 2 | 90.9 | 880.5 |
| 237 | γ-Enolase | (K)DATNVGDEGGFAPNILENSEALELVK(E) | 19.31 | 3 | 97.4 | 2702.3 |
|  |  | (K)FGANAILGVSLAVCK(A) | 18.64 | 2 | 82.4 | 1519.8 |
|  |  | (K)FTANVGIQIVGDDLTVTNPK(R) | 18.24 | 2 | 87.9 | 2102.1 |
|  |  | (K)SKFGANAILGVSLAVCK(A) | 17.24 | 3 | 84.6 | 1735.0 |
|  |  | (K)LDNLMLELDGTENK(S) | 16.95 | 3 | 92.1 | 1604.8 |
|  |  | (R)LGAEVYHTLK(G) | 16.69 | 3 | 96.0 | 1130.6 |
|  |  | (R)AAVPSGASTGIYEALELR(D) | 16.04 | 3 | 79.1 | 1804.9 |
|  |  | (K)AVDHINSTIAPALISSGLSVVEQEK(L) | 15.13 | 3 | 77.3 | 2578.4 |
|  |  | (R)GNPTVEVDLYTAK(G) | 15.12 | 2 | 86.4 | 1406.7 |
|  |  | (K)LAMQEFMILPVGAESFR(D) | 14.53 | 2 | 84.2 | 1939.0 |
|  |  | (K)ACNCLLLK(V) | 13.23 | 2 | 83.5 | 991.5 |
|  |  | (R)IEEELGDEAR(F) | 12.36 | 2 | 71.9 | 1160.5 |
|  |  | (K)LAQENGWGVMVSHR(S) | 11.56 | 2 | 71.3 | 1583.8 |

The sequence of the different peptides identified by LC-MS/MS are given for each spot together with the corresponding protein. Residues in parentheses at both ends of each peptide were inferred from tryptic digestion, and lowercase m stands for oxidized Met. For each peptide the MS/MS score is given together with its charge (z) and its scored peak intensity (SPI). The last column shows the monoisotopic mass (MH^+^) of each peptide.

**^a^**Peptides identified by *de novo* sequencing using the Sherenga algorithm.
